# Gold nanoparticle delivery to solid tumors: a multiparametric study on particle size and the tumor microenvironment

**DOI:** 10.1186/s12951-022-01727-9

**Published:** 2022-12-09

**Authors:** Mukaddes Izci, Christy Maksoudian, Filipa Gonçalves, Lucia Aversa, Robbe Salembier, Ara Sargsian, Irati Pérez Gilabert, Tianjiao Chu, Carla Rios Luci, Eduardo Bolea-Fernandez, David Nittner, Frank Vanhaecke, Bella B. Manshian, Stefaan J. Soenen

**Affiliations:** 1 NanoHealth and Optical Imaging Group, Department of Imaging and Pathology, KU Leuven, Herestraat 49, B3000 Louvain, Belgium; 2 Translational Cell and Tissue Research Unit, Department of Imaging and Pathology, KU Leuven, Herestraat 49, B3000 Louvain, Belgium; 3 Atomic and Mass Spectrometry—A&MS Research Group, Department of Chemistry, Ghent University, Campus Sterre, Krijgslaan 281-S12, 9000 Ghent, Belgium; 4 Laboratory for Molecular Cancer Biology, VIB-KU Leuven, Herestraat 49, B3000 Louvain, Belgium; 5 Faculty of Medical Sciences, Leuven Cancer Research Institute, KU Leuven, Herestraat 49, B3000 Louvain, Belgium

**Keywords:** Nanomedicine, Nanoparticles, Biodistribution, Targeted delivery, Oncology

## Abstract

Nanoparticle (NP) delivery to solid tumors remains an actively studied field, where several recent studies have shed new insights into the underlying mechanisms and the still overall poor efficacy. In the present study, Au NPs of different sizes were used as model systems to address this topic, where delivery of the systemically administered NPs to the tumor as a whole or to tumor cells specifically was examined in view of a broad range of tumor-associated parameters. Using non-invasive imaging combined with histology, immunohistochemistry, single-cell spatial RNA expression and image-based single cell cytometry revealed a size-dependent complex interaction of multiple parameters that promoted tumor and tumor-cell specific NP delivery. Interestingly, the data show that most NPs are sequestered by tumor-associated macrophages and cancer-associated fibroblasts, while only few NPs reach the actual tumor cells. While perfusion is important, leaky blood vessels were found not to promote NP delivery, but rather that delivery efficacy correlated with the maturity level of tumor-associated blood vessels. In line with recent studies, we found that the presence of specialized endothelial cells, expressing high levels of *CD276* and *Plvap* promoted both tumor delivery and tumor cell-specific delivery of NPs. This study identifies several parameters that can be used to determine the suitability of NP delivery to the tumor region or to tumor cells specifically, and enables personalized approaches for maximal delivery of nanoformulations to the targeted tumor.

**Graphical Abstract:**

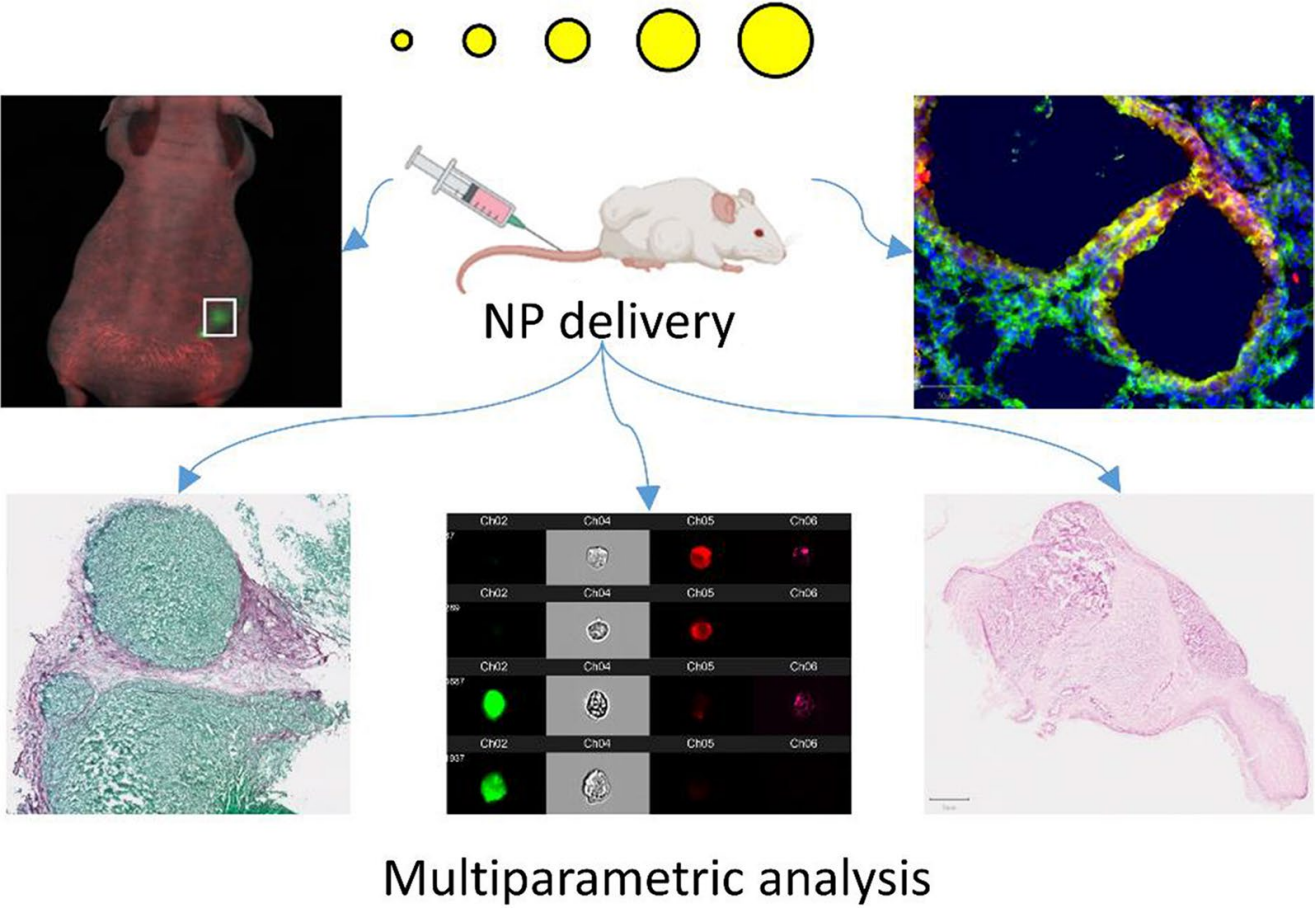

**Supplementary Information:**

The online version contains supplementary material available at 10.1186/s12951-022-01727-9.

## Introduction

The field of nanomedicine is rapidly advancing, where various formulations are undergoing clinical trials [1], and the most notable recent examples include COVID-19 vaccines [2]. Most (pre)clinical uses of nanomaterials (NMs) lie in the field of oncology, where the main application lies in the use of NMs as carriers for more common chemotherapeutic agents [1, 3]. Owing to their physicochemical properties, NMs can enhance specific delivery of any pharmaceutical agent to the tumor, either passively or by stimulated (externally triggered) release [3]. Delivery of NMs to the tumor site has long been linked to a process called enhanced permeability and retention (EPR), a process initially described in 1986 [4, 5]. For EPR to occur, NMs surfaces are then optimized for long term circulation (e.g. by addition of poly-(ethylene glycol)) in order to avoid rapid clearance by the reticuloendothelial system and this increases the chances for NMs to extravasate via the leaky endothelium of the tumor [6]. This typically results in low levels of NM accumulation in the tumor site, thus most studies make use of active targeting ligands (e.g. antibodies, peptides or membranes from host cells) to increase tumor targeting [7]. However, recent work has brought the entire concept of EPR-based targeting into question, and many factors related to NM delivery to solid tumors remain unclear [8, 9].

The meta-analysis by Chan’s group showed that in preclinical models only 0.7% of the intravenously administered dose of NMs accumulates in solid tumours irrespective of whether this occurred via passive or active targeting [10]. There has been some debate on how these data should be presented and interpreted, however, where others have argued that conventional pharmacokinetics should be used (e.g. area under the time concentration curves (AUC) for tumor versus plasma) [11]. However, while this is quite suitable for small molecules that, hopefully, result in diffusion across membranes, for NMs that are actively and rapidly cleared from the blood by the RES, even very low NM delivery levels to the tumor can rapidly result in AUC ratios. The same data expressed in this way revealed a 40% higher tumor level than plasma levels of the NMs) [11], this indicates that interpreting delivery efficacy data requires careful consideration of the most suited method used and ability to compare any data obtained with literature data. To this end, the % injected dose would provide a strong and powerful value that has been frequently used in past studies and focusses on the total level of NMs present in the tumor, therefore also indicating the high level of NMs that end up elsewhere (typically in RES organs) which should then be carefully checked for toxicity.

NM therapeutic efficacy is therefore also somewhat difficult to interpret compared to free drugs or other nanoformulations as typically different parameters will be used to describe the various entities. While 0.7% may not sound very convincing this value in itself is higher than the values obtained for many conventional drugs not associated with a nano-formulation [12]. Additionally, nano-formulations have been shown to dramatically enhance the time frame of tumor exposure by significantly reducing the clearance rate of the agent [12]. Nano-formulations have therefore been shown to have great clinical potential, but also have a large window of opportunity for further improvement by boosting the delivery efficacies [10].

Various efforts have recently been undertaken to try and improve our understanding of how NMs are delivered to solid tumors [7]. A recent study using mice unable to perform active transcytosis revealed that NM delivery dropped significantly, suggesting that the contribution of passive NM diffusion through leaky tumoral blood vessels was negligible [13]. Another study revealed a dose-limiting barrier for efficient delivery, where for mice, at least a trillion NMs are required which will result in saturation of the RES system and phagocytic cells, and therefore result in higher levels of NM reaching the tumor [14]. Other studies revealed that while NMs may reach the tumor site, the number of tumor cells that will take up any NMs is extremely low, where most NMs will be stuck in the extracellular matrix or tumor-associated macrophages [15]. Overall, these data highlight the need for more in-depth analysis of NM tumor delivery to try and understand better the mechanisms involved which will hopefully result in better tumor targeting methods.

Apart from differences in NM properties, it has become clear that efficient delivery of NMs is strongly associated to tumor physiology. The high level of variability in tumor-related parameters will complicate any straightforward analysis of NM delivery [16]. One trend that has emerged is the need for personalized medicine, where depending on the physiology of the tumor of a particular individuum, this tumor would be more or less susceptible for NM therapy [6].

The current study aims to address these challenges by studying the delivery efficacy of a series of differently sized gold NMs in view of a multitude of tumor-associated parameters. Using dimensionality reduction analysis we then try and define which parameters govern NM delivery.

## Results

### Nanomaterial characterization

In the present study, gold nanoparticles (Au NPs) were used as the NM type of choice, owing to multiple factors, including: (1) the chemical inertness of Au NPs ensuring that we measure complete NPs as no in vivo degradation of Au NPs occurs; (2) the high degree of chemical control over Au NP synthesis, enabling tight control over NP surface characteristics and size and (3) the ability to accurately quantify Au NP amounts by means of inductively coupled plasma mass spectrometry (ICP-MS) [15].

A total of 5 differently sized NPs were used, being 10, 20, 40, 60 and 80 nm in diameter (core diameter). All NPs were coated with poly(metacrylic acid) and 2 kDa methoxy-poly(ethylene glycol) (PEG) linked to AF647. NP core sizes were evaluated using transmission electron microscopy (TEM), the hydrodynamic diameter and surface charge were measured using dynamic light scattering and zeta-potential measurements while colloidal stability of the NPs was evaluated using nanoparticle tracking analysis.

**Fig. 1:**
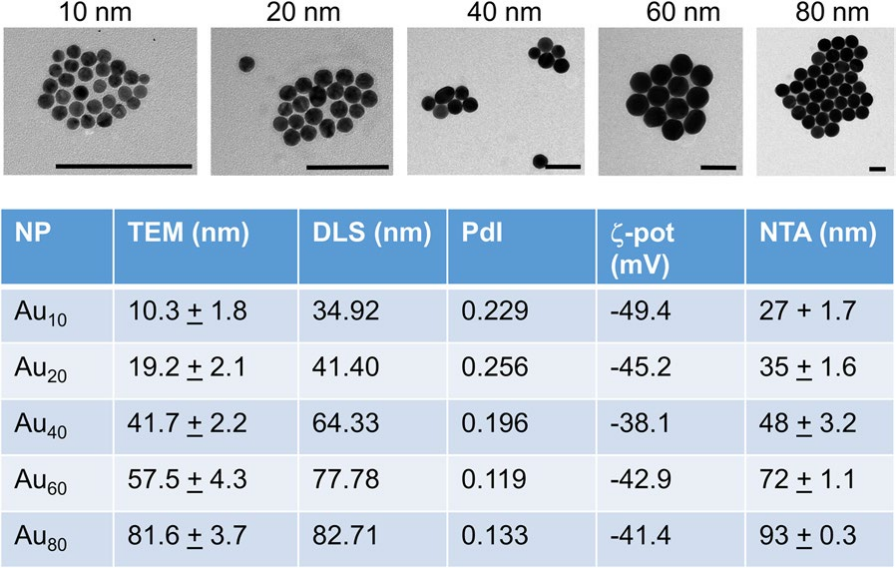
Nanoparticle characterization data. Representative transmission electron micrographs are given for the 5 differently sized gold nanoparticles that are used in this study. Scale bar in every image is 100 nm. The table below show various parameters for every nanoparticle used. The first parameter is the core diameter (determined by TEM) and analysed by measuring 100 NPs over different images. The second parameter is the hydrodynamic diameter in aqueous environment (determined by DLS in PBS). The third parameter is the polydispersity index (PdI), determined simultaneously by DLS which indicates colloidal stability of the NPs in PBS. The fourth parameter is the ζ-potential, which is the NP surface charge, as measured in PBS. The fifth parameter is the colloidal stability of the NPs in high levels of serum (as determined by NTA in 50% FBS-containing PBS)

**Fig. 2:**
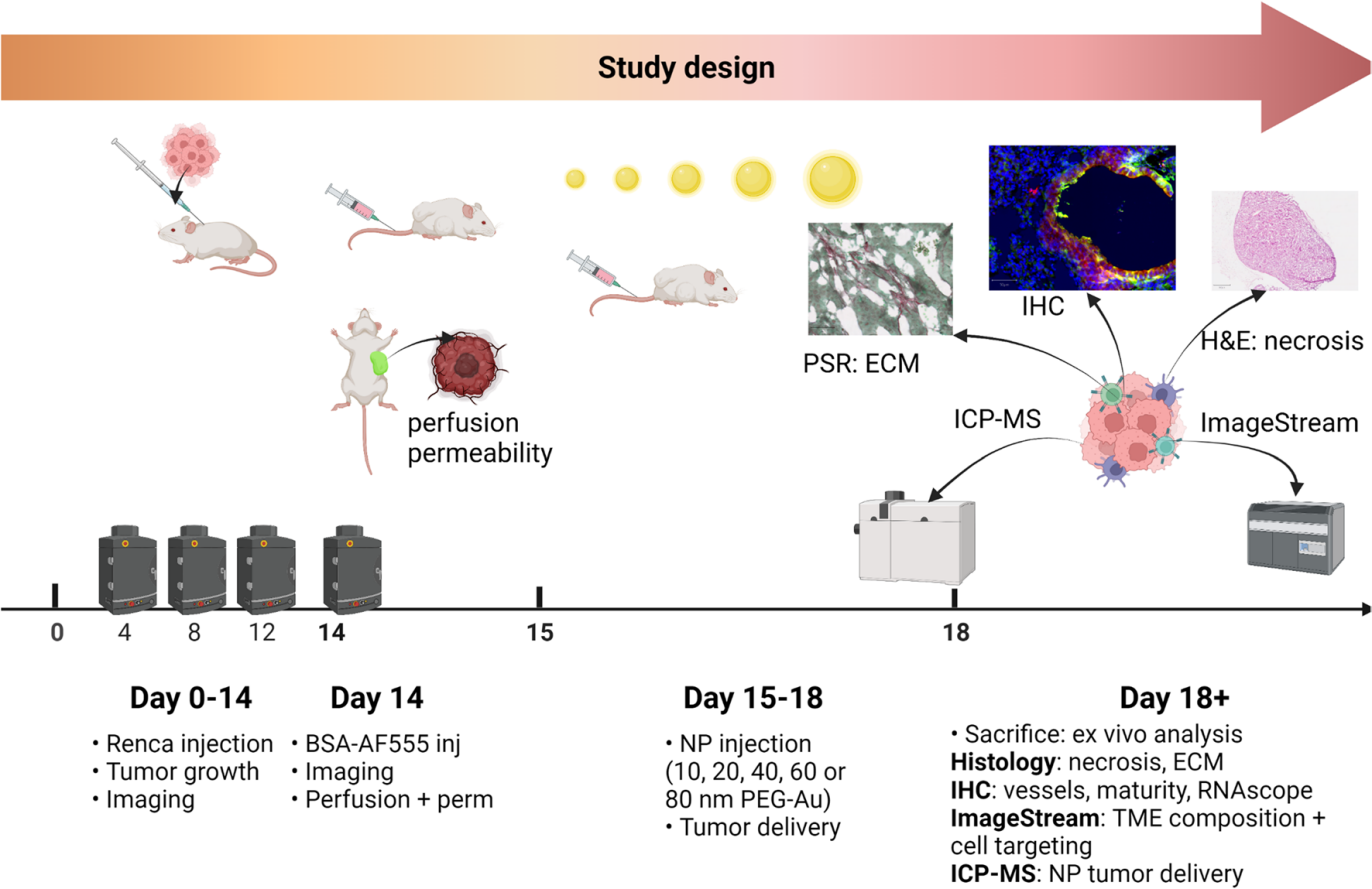
Schematic overview of the study design. Upon subcutaneous administration of luminescently-tagged Renca cells, non-invasive imaging was performed at days 4, 8 and 12 to monitor tumor growth. At day 14, AF555-tagged BSA was administered intravenously and optical imaging was performed to determine relative perfusion and permeability of tumor-associated blood vessels. At day 15, animals received 100 µL of PBS with Au NPs (150 µg Au/mouse, either 10, 20, 40, 60 or 80 nm diameter in size) by intravenous administration. The NPs were then left to circulate for 72 h, after which time we had observed a complete absence of the NPs in the blood, after which the animal and the tumors were analyzed ex vivo for potential toxicity, NP tumor delivery efficacy, and tumor-associated parameters as indicated in the image

Figure 1 provides an overview of NP characteristics, revealing a good and narrow size distribution of the NPs close to their theoretical sizes. All NPs possessed a negative surface charge and were on average 20 nm larger in size due to the presence of the PEG chains and immobile unit of the solvent ions. Colloidal stability of all NP formulations under physiological (50% serum) containing conditions was good (nanoparticle tracking analysis), after which the NPs themselves were used in tumor-bearing mouse models as depicted in Fig. 2.

### Administration of Au NPs to tumor-bearing mice

**Fig. 3:**
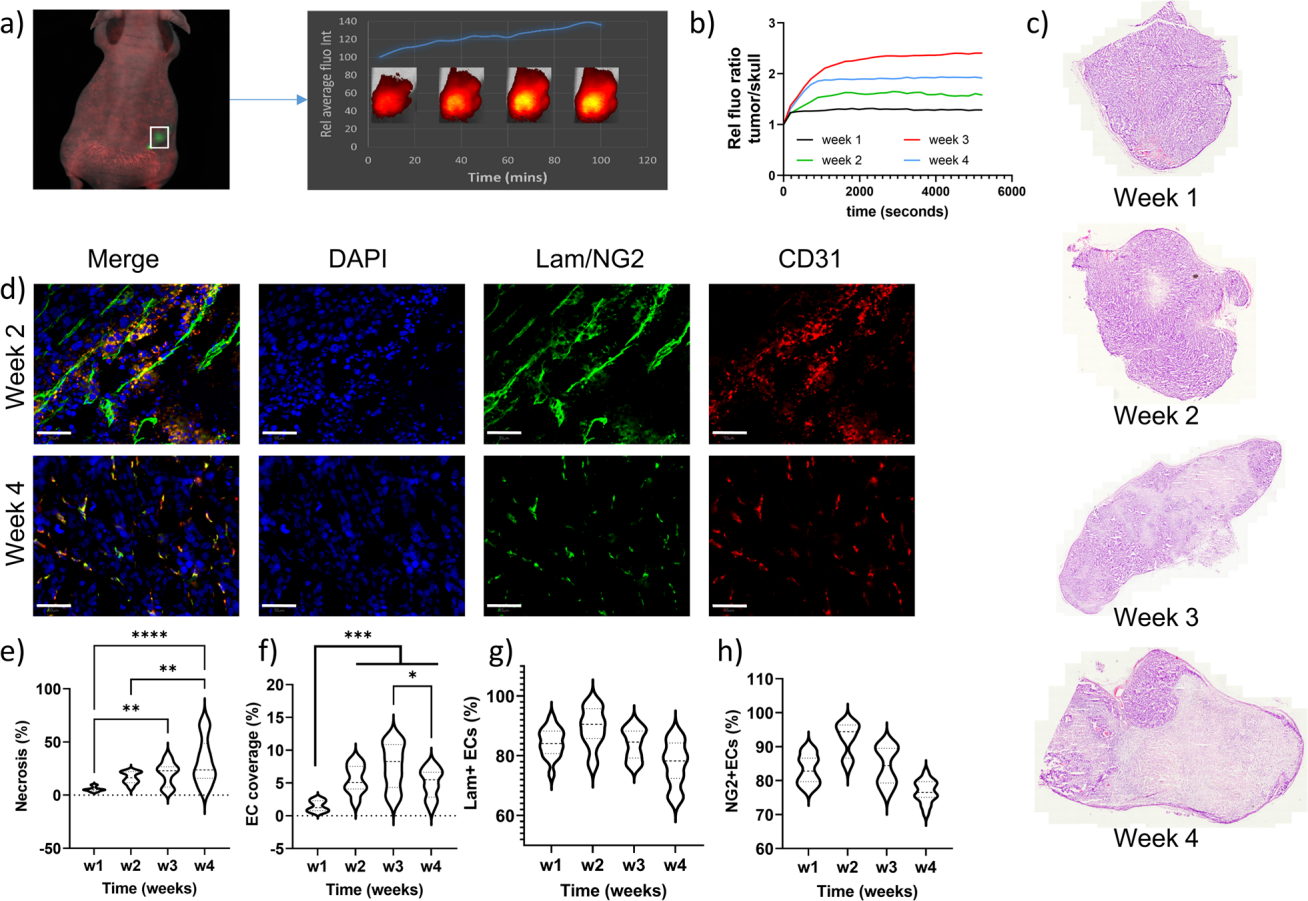
The effect of tumor growth on perfusion, vessel maturity and necrosis. **a** Representative image of a Renca-bearing mouse, showing tissue background signal (red) and Renca cell-specific GFP (green) for selection of the ROI. Upon administration of AF555-BSA, the fluorescent signal of BSA was measured repeatedly over time (right image) and fluorescence intensity could be measured and expressed as function of time. **b** The relative ratio of AF555-BSA signal of the tumor over the same ROI placed on the skull of the animal. The data show average data pooled from at least 6 animals per group. **c** Representative H&E-stained tumor sections obtained 1, 2, 3 or 4 weeks after tumor engraftment. **d** Representative immunohistochemistry images of tumor tissue sections stained with anti-CD31 (endothelial cells), anti-laminin (top image: basal membrane), NG2 (pericyte marker: bottom image), and counterstained with DAPI nuclear stain. The top row shows images of tumor section obtained 2 weeks post engraftment, the bottom row at 4 weeks post engraftment. The scale bar is 50 µm. **e–h** Violin plots indicating the level of e) necrosis, f) relative endothelial cell area, g) the percentage of laminin-covered vessels, and h) the percentage of NG2-covered vessels as a function of time. Significant differences in necrosis and endothelial cell coverage is indicated where appropriate (n = 15; *: p < 0.05; **: p < 0.01; ***: p < 0.001; ****: p < 0.0001)

Firefly luciferase-expressing renal carcinoma cells (Renca) were used in this study, as a well-developed and commonly used syngeneic tumor model. Renca cells were implanted subcutaneously, after which tumors were allowed to grow. The perfusion and blood vessel permeability of the mice was evaluated using a high-throughput optical imaging setup, where fluorescently labeled bovine serum albumin (AF555-BSA) was administered intravenously to the mice and the kinetics of BSA distribution was evaluated for 90 min (Fig. 3a). Perfusion and blood vessel permeability were then determined as relative units compared to the skull, where no leaky blood vessels would be present. Initial data over a 4 week period revealed that in the first week after injection, some tumor growth occurred, but blood vessel formation was minimal, with limited perfusion in the tumor (Fig. 3b).

In the 2nd and 3rd week following tumor cell administration, maximal values for perfusion and permeability were observed, which then decreased again after 4 weeks. The latter is likely due to the ongoing growth of the tumor itself, which results in higher levels of necrosis and extracellular matrix formation and hereby hinders efficient perfusion of the larger tumors [17]. This was confirmed by ex vivo analysis of tumors studied at weekly intervals post engraftment (Fig. 3c–h), where tumor necrosis was found to increase with time (Fig. 3c, e). The total blood vessel coverage reached a maximum at 3 weeks (Fig. 3d, f), while basal membrane coverage and vessel maturity reached maxima after 2 weeks (Fig. 3d, g, h). We therefore selected the period between 2–3 weeks as the optimal frame to start the study, with initial BSA studies at 14 days post tumor cell engraftment, followed by NP administration at day 15 and analysis at day 18 (Fig. 2). In total, approximately 20 mice were used per group (size of NPs). In short, 2 weeks following tumor cell administration, tumor growth, perfusion and blood vessel permeability were evaluated by non-invasive optical imaging. The next day, Au NPs were administered intravenously as a single bolus (all NPs were administered at the same mass of gold), after which the animals were kept for 72 h and then sacrificed for analysis. The time of 72 h was chosen as the time by which no Au could be found in blood samples and therefore all NPs were expected to have extravasated.

### Tumor-related parameters in the study group

**Fig. 4:**
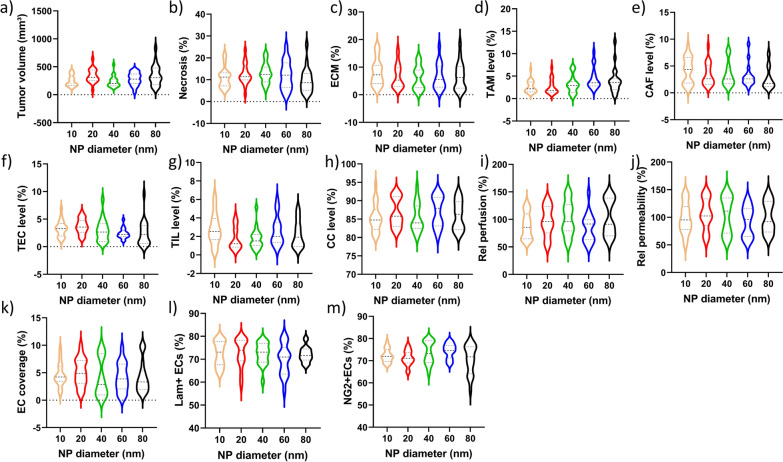
Evaluation of tumor-specific parameters across the different groups. Violin plots of the respective indicated parameter for every group of animals (n > 16 per NP size). **a** The tumor volume, expressed in mm^3^ and measured by caliper measurements immediately prior to tumor resection. **b** The area of necrotic tissue in the tumor as determined by H&E staining relative to the area of the entire tissue slice. **c** The area positive for ECM in the tumor as determined by Picrosirius Red staining relative to the area of the entire tissue slice. **d–h** Relative level of d) TAMs, e) CAFs, f) TECs, g) TILs and h) cancer cells (CC) expressed relative to the total number of cells as determined by ImageStreamX Mark II analysis. **i**, **j** Relative level of **i** tumor perfusion and **j** tumor vessel permeability determined by AF555-BSA mediated optical imaging and expressed relative to the perfusion and permeability of the skull in the same mice. **k–m** The relative level (%) of k) tumor area occupied by CD31^+^ endothelial cells, l) CD31^+^ blood vessels covered by laminin, m) CD31^+^ blood vessels covered by NG2

The tumors themselves were analyzed for a variety of parameters including size, level of necrosis, the density and extent of the extracellular matrix, the level of tumor-associated macrophages (TAMs), cancer-associated fibroblasts (CAFs), tumor-infiltrating lymphocytes (TILs), tumor-associated endothelial cells (TECs), cancer cells, the perfusion of the tumor by blood vessels, the maturity of the blood vessels and their coverage by basement membrane and pericytes (Fig. 4, Additional file 1: Figures S1–S6). Data across all groups revealed that while individual values for particular parameters could vary, no significant differences were observed between different groups, exposed to differently sized Au NPs.

All values obtained were within physiologically relevant values, where the average tumor size of 366 mm^3^ is far below ethical considerations yet sufficiently large for therapeutic considerations [18]. Tumor necrosis levels were very valuable, but did not appear to be linked to tumor size itself. However, the relatively high level of necrosis correlates with the aggressive nature of the Renca model system [19]. The level of ECM present is quite normal in view of the size of the tumor, where ECM density typically correlates with tumor size [20]. On the other hand, as collagen has a dual role in tumor progression and collagen remodeling plays a major role in tumor growth and migration, the aggressive nature of the Renca tumor model will also influence ECM density [21]. The relatively low levels of TAMs, CAFs, TECs and in particular TILs are typical for syngeneic murine models which generally exert very low levels of immunogenicity. Of the most common studied tumor models, Renca cells are however one of the slightly more immunogenic models, therefore still resulting in considerable levels of non-tumor cells in the tumor microenvironment (TME) [22].

### Evaluation of nanoparticle delivery to solid tumors

Upon administration of the Au NPs, blood biochemistry analysis along with histopathological evaluation of all major organs did not reveal any toxicity induced by the NPs at the concentrations used (Additional file 1: Figures S7, S8). When looking at the overall level of Au NPs that reached the tumor, the average value irrespective of NP size was 0.70% ± 0.07% (mean ± SEM; *n* = 94) of the injected dose of NPs.

**Fig. 5:**
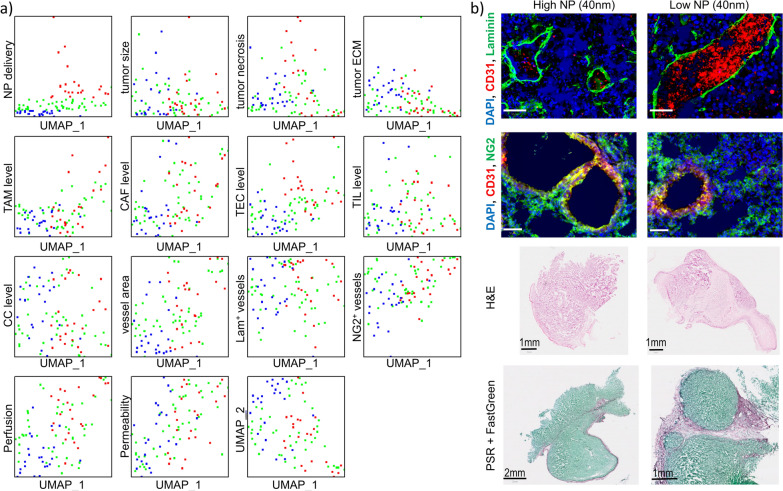
Uniform parameters that determine NP delivery efficacy across different NP sizes do not exist. **a** UMAP plots for each and every tumor-associated parameter determined (as displayed in Fig. 4) as a function of UMAP coordinates. For analysis, all data points for each and every animal were combined for all groups (all differently sized NPs were analysed together). For every parameter, the values were first rescaled to a linear 0–1 scale with 0 being the lowest value for that parameter across all animals and 1 being the highest value for that parameter across all animals. Every single dot shown reveals a separate animal and indicates the specific value of the animal on its Y-axis, while for every animal, its position on the X-axis does not change and thus, values for all parameters can be directly compared. The dots were colour-coded based on the upper left plot (the total tumor NP uptake level), where the 25% of animals with highest NP tumor levels were coloured red, the 25% of animals with lowest NP tumor levels were coloured blue and the remaining animals with medium NP levels were coloured green. To determine whether a particular parameter promotes or inhibits NP delivery efficacy, the red and blue groups should be separated on the Y-axis for the parameter. The parameters with most distinction (but not complete) were TEC level and tumor perfusion. **b** Representative images for Au_40_ NP treated mice having high (left column) or low (right column) NP delivery efficacy in the tumor. The top row show laminin-stained tumor blood vessels, indicating less laminin coverage in the low NP delivery group (scale bars: 50 µm). The second row shows NG2-stained tumor blood vessels, also indicating less pericyte (NG2) coverage in the low NP delivery group (scale bars: 50 µm). The third row shows H&E images, showing higher necrosis levels in the low NP delivery group. The fourth row shows PSR images, showing higher ECM density in the low NP delivery group

This was perfectly in line with previous studies, as described in the meta-analysis by Wilhelm et al. [10]. Further analysis of NP delivery efficacy linked to particular tumor parameters involved statistical modelling, where all parameters were first modeled on a scale from 0 (lowest value) to 1 (maximum value) in order to avoid skewing the data towards parameters with higher numerical values. Then dimensionality reduction analyses were performed, specifically being Uniform Manifold Approximation and Projection (UMAP) analysis that enables multiparametric analysis to be grouped and expressed over two dimensions to reveal similarities between groups. UMAP analysis of NP delivery to Renca tumors irrespective of NP size resulted in various groups, but detailed analysis of each and every parameter did not reveal any clear descriptors promoting either low or high NP delivery (Fig. 5a). Therefore, no generally applicable parameters could be defined that would enable one to conclude whether a tumor is generally suitable for NP delivery.

### Evaluation of NP delivery to solid tumors as a function of nanoparticle size

**Fig. 6:**
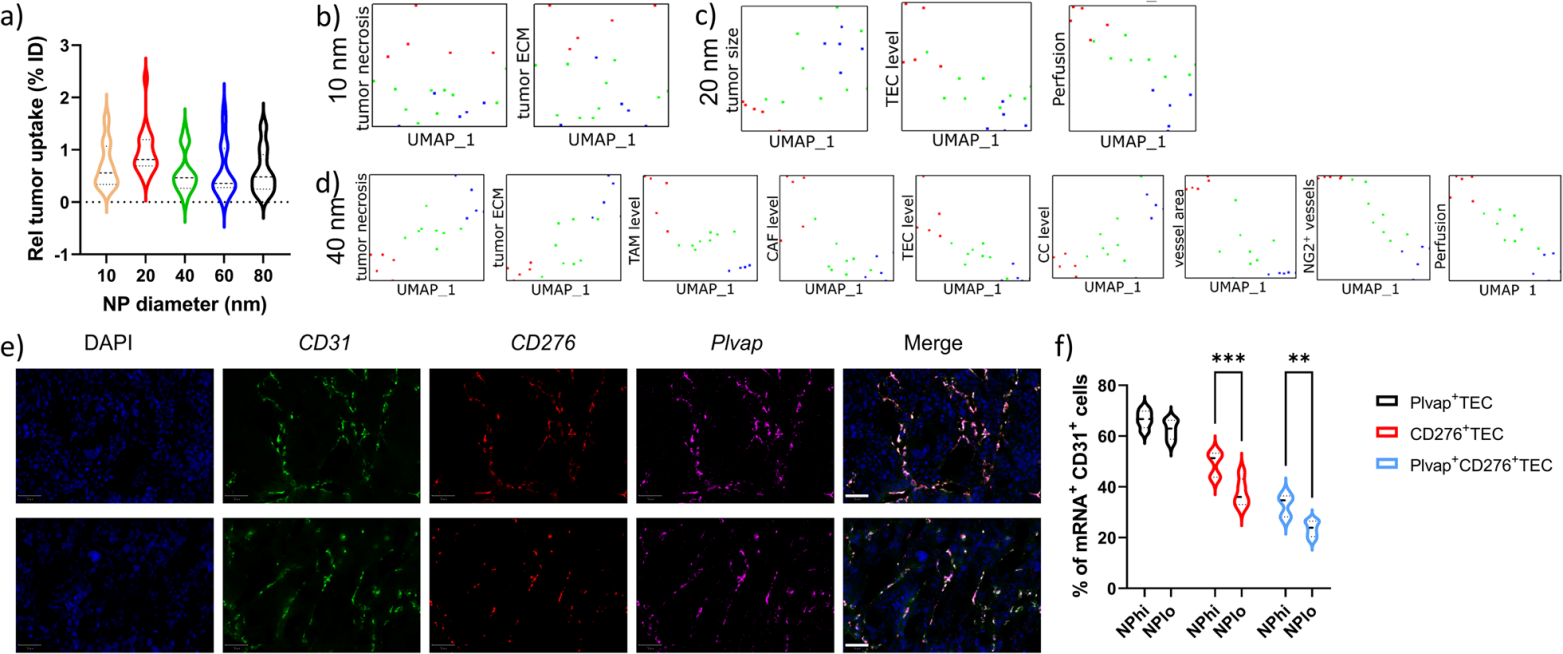
The influence of NP size on tumor-associated parameters influencing NP delivery efficacy and the role of specialized tumor-associated endothelial cells. **a** Violin plots showing the NP delivery efficacy for every group of NPs expressed as the % of tumor-associated NPs relative to the originally administered amount. **b**–**d** UMAP plots for tumor-associated parameters that were found to influence NP delivery efficacy for **b** Au_10_ NPs, **c** Au_20_ NPs and **d** Au_40_ NPs. Please note that the plots for Au_40_ NPs represent the same parameters as relevant for Au_60_ and Au_80_ NPs (a complete overview of all UMAP plots for all NP sizes can be found in Additional file 1: Figures S9–13). e) Representative RNAscope images of tumor sections stained against CD31, CD276, Plvap mRNA and counterstained with DAPI nuclear stain. Scale bars = 50 µm. The top row is from a tumor with high NP levels, the bottom row is from a tumor with low NP levels. **f** Violin plots of quantified RNAscope data expressed as the % of Plvap^+^ TECs, CD276^+^ TECs and Plvap^+^CD276^+^ TECs relative to total TEC levels for tumors with high and low NP delivery efficacy. Statistically significant differences between high and low NP groups for every parameter is indicated where appropriate (n = 10; **: p < 0.01; ***: p < 0.001)

As the lack of unified descriptors may be due to the fact that differently sized NPs were used, the same analysis was performed for every individual group of animals treated with NPs of a particular size (Fig. 5b). Looking at differently sized NPs, one surprising observation was that on average, 20 nm diameter NPs resulted in the highest tumor delivery levels, whereas no differences could be observed between any other NP sizes (Fig. 6a). The higher average NP delivery levels for 20 nm diameter Au NPs are likely linked to specific properties of the Renca tumor model and will not be general for every type of tumor. The parameters that have been most commonly linked to tumor delivery of NPs is the size-dependent transport of NPs across the vessel walls and then the transport through the tumor interstitial space [23]. In both cases, larger NPs are more hindered in their free movement, while smaller NPs can also be cleared faster and be reintroduced in the blood stream or cleared away in the lymphatic system [24]. Previous investigations by the Jain group revealed that the ideal NP size for tumor-specific extravasation was between 12 and 20 nm, but this value depends on the level of tumor vessel maturity and ECM density and will show inter- and intratumoral variation [23]. This is also apparent from our data, where on average 20 nm diameter NPs resulted in higher tumor delivery rates, but when looking at individual subjects, every group contained different subjects with better NP delivery levels than the lower half of the 20 nm diameter group.

The lower number of subjects impaired a proper grouping upon UMAP dimensionality reduction, where in most cases the individual subjects were quite spread (Fig. 6b–d). Looking into the individual parameters, clear distinctions could be made in the parameters that either promote or impede NP tumor delivery (Fig. 6b–d, Additional file 1: Figures S9–S13). The parameters themselves were furthermore also dependent on the size of the NMs themselves. For the 10 nm diameter NPs, somewhat surprisingly, a more dense ECM and higher levels of necrosis promoted NP delivery efficacy, while no other parameters played a clear role (Fig. 6b, Additional file 1: Figure S9). The higher ECM density and necrosis levels may contribute to reduced clearance of the NPs out of the tumor after extravasation. Smaller NPs tend to be removed faster out of the tumor than larger ones, and the extent of this process has been directly linked with the extent of ECM density [25]. Most surprisingly here is the fact that the extent of perfusion or endothelial cell coverage or maturity did not play any significant role at all and even poorly perfused tumors were accessible by 10 nm diameter NPs. As the physical dimension of the NP is too large to enable free diffusion of the NPs across cell membranes, the poorly perfused tumors may, together with increased ECM density and higher levels of necrosis lead to areas with reduced blood flow, in which the speed of blood flow is highly reduced and could therefore promote NP extravasation, similarly as what happens in the liver compared to more perfused tumors [26]. This effect could be further augmented by the proposed ability of the Au NPs to induce endothelial leakiness by disrupting the VE-cadherin–VE-cadherin homophilic interactions at the adherens junctions, as has been described for Au NPs in breast tumor models [27].

For 20 nm diameter NPs, ECM density and necrosis no longer played a role, but delivery was correlated with smaller tumor size, higher levels of endothelial cells and perfusion (Fig. 6c, Additional file 1: Figure S10). The lack of distinct effects of necrosis and ECM density likely indicate that the size of these NPs is ideal for initial tumor delivery in the Renca model, where it is small enough to extravasate and move through the interstitial tissue, while not too large to become easily trapped within the ECM. The importance of perfusion and higher levels of endothelial cells is in line with expectations for an easy delivery of the NPs, yet the maturity of the blood vessels did not play a role for these NPs, which suggests that the NPs did not primarily extravasate through endothelial gaps. The contribution of the small size of the tumor is somewhat unclear, but this may be assigned to the readout parameters used. The amount of NPs delivered is expressed per g/tissue, and therefore larger tumors will require larger amounts of gold to delivered. Furthermore, as perfusion is an important contributing factor, smaller tumors are more likely to be overall well perfused as larger tumors likely consist of heterogeneous regions of poor and good perfusion [28].

For NPs of 40 nm and above, more similarities were observed (Fig. 6d, Additional file 1: Figures S11–13), where overall higher necrosis and ECM density impaired NP delivery, but higher levels of perfusion, endothelial cells coverage, maturity of blood vessels, levels of TAMs and CAFs all promoted higher NP delivery levels. While for 20 nm diameter NPs, necrosis and ECM density did not play a distinct role, the positive contribution observed for both parameters in the case of 10 nm NPs has been completely reversed into an inhibitory function. These effects are in line with the classical view of EPR, where necrotic regions are less perfused, and high ECM density prevent NP extravasation and transport of the NPs throughout the interstitial tissue. The high level of perfusion logically aids in delivering the larger NPs as larger blood volumes will increase the number of NPs reaching the tumor site and their chances of extravasation. The influence of TAMs are in line with expectations as well, where TAMs have been frequently described to take up NPs reaching the tumor site [29]. The influence of CAFs is somewhat more surprising, as higher CAFs levels are typically linked to higher ECM densities. However, as the nature and heterogeneity in CAFs functions are very broad and currently not fully understood [30], high levels of CAFs in the absence of increased ECM density has a positive effect on NP delivery.

Another surprising finding is that while perfusion and vessel coverage in the tumor contribute to NP delivery, in support of EPR effects, vessel permeability did not play a particular role at any level, and mature vessels even contributed more to NP delivery. The latter may be due to the reduced interstitial pressure associated with mature vessels compared to leaky structures, which is known to enhance for example chemotherapy influx into tumors [31]. An alternative possibility lies in the presence of so-called nanoparticle transport endothelial cells (N-TECs) that have recently been described as being the type of endothelial cells that contribute to NP uptake [32]. To study this, *Plvap* and *CD276* expression levels were evaluated using RNAScope technology on the 5 tumors with highest and 5 tumors with lowest NP levels (Fig. 6e, f). The markers *CD276* and *Plvap* were selected based on the study by Kingston and colleagues, who described these two genes as being significantly upregulated in N-TECs compared to other endothelial cells [32]. The data obtained by the RNAScope analyses support these findings and our hypothesis. Both markers showed a clear correlation of expression level and respective NP internalization amounts. This reached significant differences in *CD276*^+^ TECs and *CD276*^+^*Plvap*^+^ TECs. Interestingly, as the data were obtained for differently sized NPs, they were uniformly valid and could potentially serve as proper models for predicting NP tumor delivery efficacy, instead of classical perfusion/permeability tests of tumors to obtain information on the ability of the tumor to be used for NP-based treatments.

### Evaluation of NP delivery to tumor cells

**Fig. 7:**
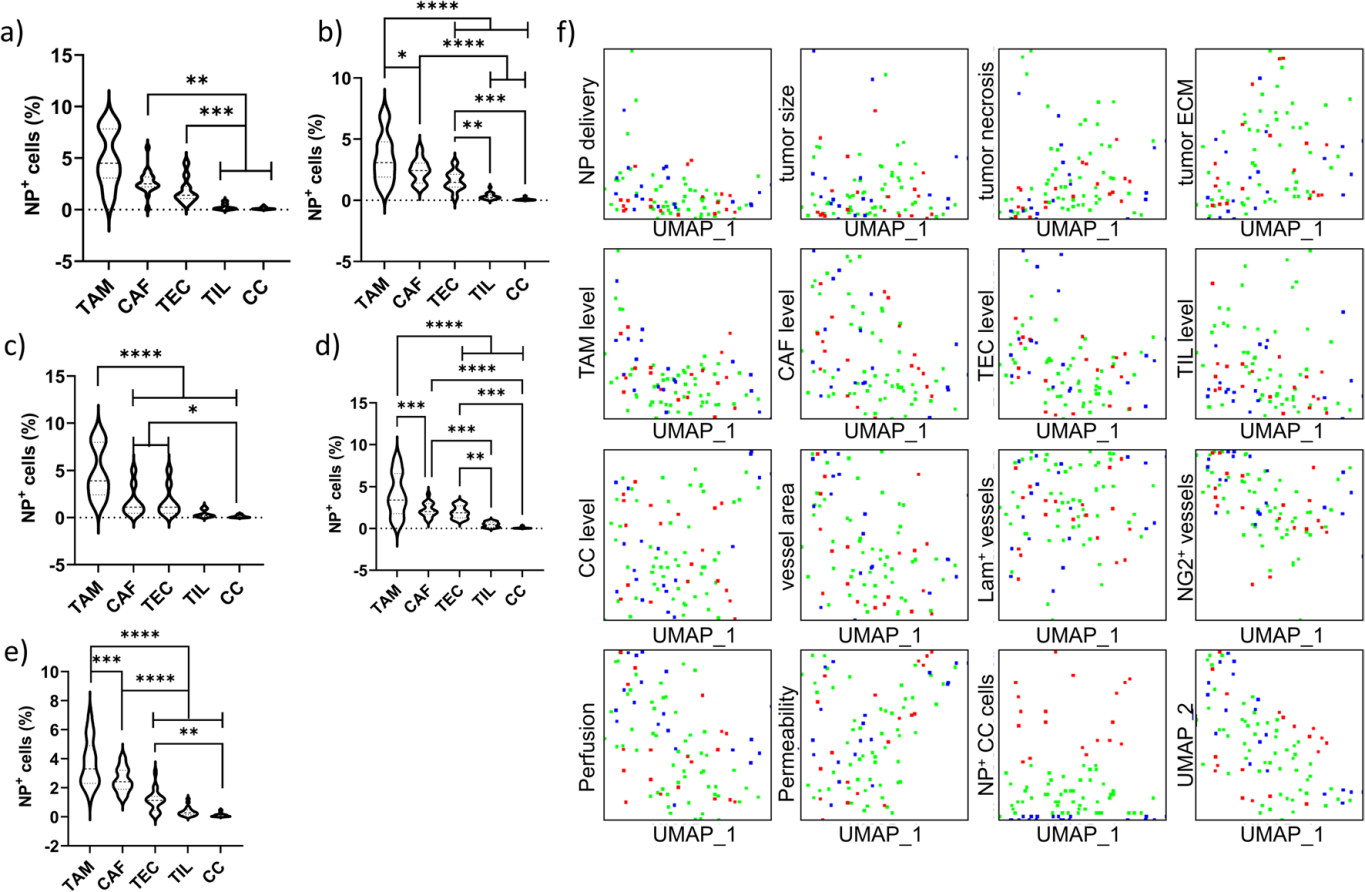
NP delivery to tumor results primarily in TAM and CAF association and poor uptake by tumor cells. **a**–**e** Violin plots showing the relative percentage of NP^+^ TME-related cell types expressed as the % of NP^+^ cells as determined by ImageStreamX Mark II expressed relative to the total gated cell type. The data are shown for **a** Au_10_ NPs, **b** Au_20_ NPs, **c** Au_40_ NPs, **d** Au_60_ NPs, **e** Au_80_ NPs. **f** UMAP plots for each and every tumor-associated parameter determined (as displayed in Fig. 4) as a function of UMAP coordinates. For analysis, all data points for each and every animal were combined for all groups (all differently sized NPs were analysed together). For every parameter, the values were first rescaled to a linear 0–1 scale with 0 being the lowest value for that parameter across all animals and 1 being the highest value for that parameter across all animals. Every single dot shown reveals a separate animal and indicates the specific value of the animal on its Y-axis, while for every animal, its position on the X-axis does not change and thus, values for all parameters can be directly compared. The dots were colour-coded based on the level of NP^+^ cancer cells (2^nd^ to the right of the bottom), where the 25% of animals with highest NP tumor levels were coloured red, the 25% of animals with lowest NP tumor levels were coloured blue and the remaining animals with medium NP levels were coloured green. To determine whether a particular parameter promotes or inhibits NP delivery efficacy, the red and blue groups should be separated on the Y-axis for the parameter

Apart from delivery NPs to the tumor site, the number of nanoformulations that specifically interact with cancer cells, rather than with any other cell type in the TME is of high importance. We therefore analysed all the tumors with image-based cytometry, using antibody sets to determine cancer cells (CD45^−^CD24^+^), tumor-associated macrophages (TAMs; F4/80^+^), cancer-associated fibroblasts (CAFs; CD45^−^CD90.2^+^), tumor-associated endothelial cells (TECs; CD144^+^) and tumor infiltrating lymphocytes (TILs; CD45^+^CD3^+^) (Additional file 1: Figure S14). The data for all tumors reveal the highest relative level of NP uptake to occur in TAMs and CAFs, followed by TECs and lastly by cancer cells and TILs (Fig. 7a–e). While the high uptake of NPs by TAMs has been well documented, the contribution of CAFs in NP uptake is somewhat surprising. CAFs are regarded as major contributors to the TME and tumor progression and have been the subject of targeted therapy developments, including the use of nanoformulations [17]. The data here reveal that CAFs are inherently prone to targeted delivery by NPs, which could be further improved by the use of pharmacological treatments to induce CAF depletion [33].

The low level of cancer cell involvement is in line with other studies, while the level of cancer cells being exposed to NPs on average is higher in our study than in the one described by the Chan group [15]. This may be due to differences in detection methods, where in the study by Dai and colleagues, fluorescence signals were used to evaluate NP levels by means of flow cytometry [15]. Depending on the need for compensation of fluorescence signals, this may hinder the detection of low levels of NPs in cancer cells. In our study, we used image-based flow cytometry, which aids in determining intracellular localization of the NPs by the ability to generate masks of the cells and only taking NP signals into account that are located within this mask [34]. Furthermore, detection occurred via dark field imaging, which helps in reducing the need for compensation and may further influence detection sensitivity. Another factor lies in the differences between tumor types. The distribution of NP across various cell types will depend on the level of those cell types in the TME. For tumors with higher levels of TAMs, this will automatically result in lower levels of cancer cells being exposed to the NPs themselves. Any comparison between different studies would therefore require an in-depth investigation of the TME composition, preferably using standardized methods.

### The impact of tumor physiology on the delivery of NPs to tumor cells

Similar as for the delivery of NPs to the tumor itself, we set out to define which parameters play a role in the delivery of NPs to cancer cells. Using the dimensionality reduction approach described earlier for the entire population of NPs, the level of TAMs and CAFs were found to both play a minor inhibitory role, seemingly irrespective of NP size (Fig. 7f). This is in line with our observation above, where the high level of NP uptake by TAMs and CAFs would impede NP delivery to cancer cells. No other factors immediately stood out, and therefore the dimensionality reduction analysis was also performed for each and every size of NP (Additional file 1: Figures S15–S19).

For the 10 nm diameter NPs, no single parameter played any distinct role in determining the delivery efficacy to tumor cells (Additional file 1: Figure S15). Even the presence of TAMs or CAFs did not appear to be a major hindrance to the delivery of the Au NPs to the solid tumors, but overall, those tumors that had relatively higher levels of tumor cells containing NPs, were tumors that on average had lower overall NP levels. For 20 nm diameter NPs, no distinct parameters were present either, but a tendency for low TAM levels and higher levels of blood vessels (combined with high levels of maturity; maturity levels alone were not sufficient if the total number of vessels was low) resulted in an increase in tumor cell NP uptake (Additional file 1: Figure S16). These findings are in line with our reports above, where higher TAM levels will scavenge the NPs before they can reach the tumor cells themselves, while high levels of mature blood vessels (high in number and in maturity) supports our the data on so-called N-TECs as specialized cells that play an important role, not only in increasing overall NP tumor delivery [32], but also on delivery to tumor cells specifically.

For 40, 60 and 80 nm diameter NPs, the results are again quite similar to each other (Additional file 1: Figure S17–19), where low TAM, CAF and TEC levels are required to reach higher levels of NP-containing tumor cells. The low levels of TECs are conflicting with the results for overall NP levels and the results obtained with 20 nm diameter NPs. While the presence of N-TECs in these tumors do still help in increasing overall NP levels, as shown earlier, this effect is not observed for tumor cell-specific uptake. It may be that high levels of N-TECs require the presence of CAFs and TAMs as all members of the TME interact closely with one another [35]. Alternatively, as low TEC levels here also promote tumor cell-specific NP association levels, the overall level of N-TECs will therefore also be reduced compared to tumors with higher number of blood vessels. For the ‘bigger’ NPs, the internalization of NPs by TECs seems to counter the improved delivery to tumor cells, suggesting that the activity of NP transport across the endothelial barrier through N-TECs may exhibit size-dependent limitations.

While the data for tumor-cell specific targeting is important to consider in improving targeted NP delivery strategies, it is also important to note the limitations of this analysis, where in the final part, “tumor cell targeting”, reflects those cells that in relative terms, had the highest proportion of NP-positive cells. However, as the level of NP-positive tumor cells is very low, any differences in these levels may not always be attributed to any distinct parameter as the overall impact is simply too low. Furthermore, while the level of NP-positive tumor cells may be higher, the overall NP uptake level is always on the lower end. Therefore, while in other tumors with more TECs, TAMs or CAFs, the relative level of NP-positive tumor cells may be slightly lower, this is potentially compensated by higher levels of NPs overall.

**Fig. 8:**
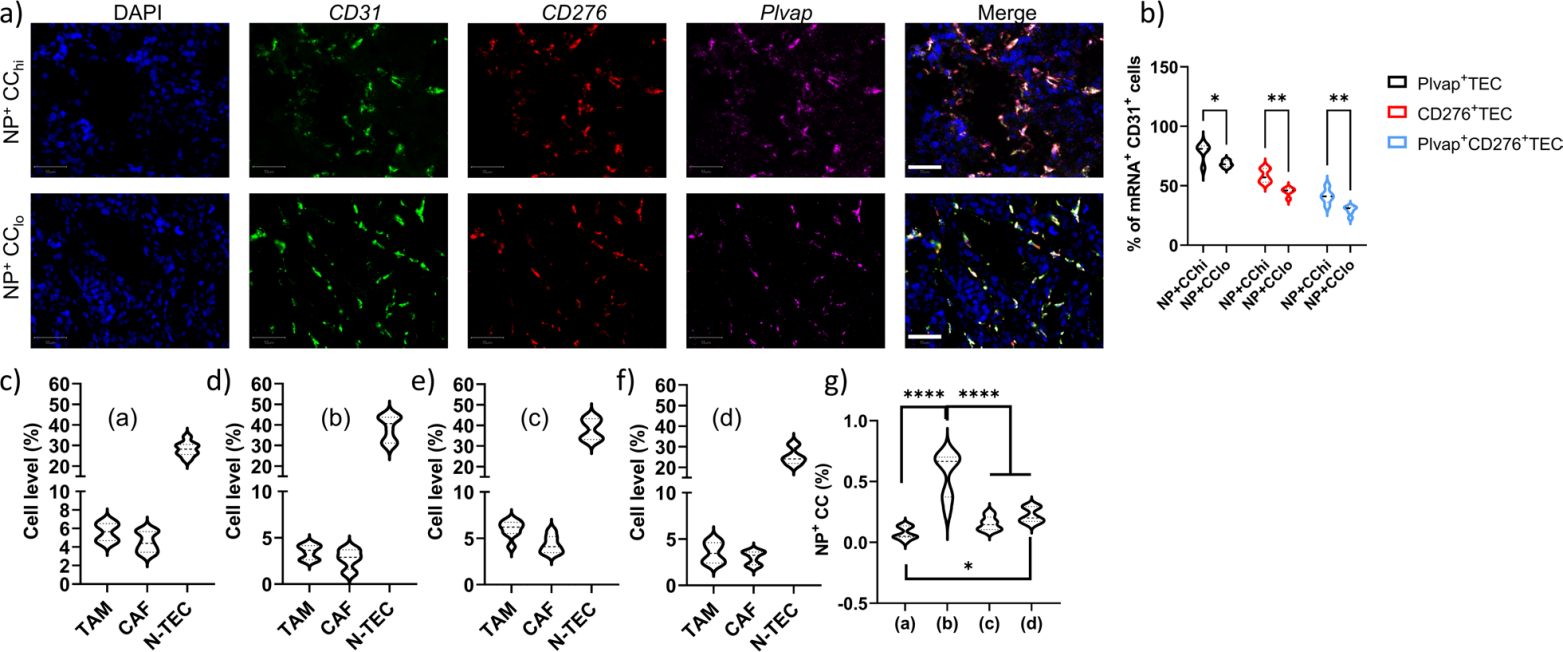
Tumor cell specific NP delivery is linked to specialized TECs, TAM and CAF levels. **a** Representative RNAscope images of tumor sections stained against CD31, CD276, Plvap mRNA and counterstained with DAPI nuclear stain. Scale bars = 50 µm. The top row is from a tumor with high levels of NP^+^ cancer cells, the bottom row is from a tumor with low levels of NP^+^ cancer cells. **b** Violin plots of quantified RNAscope data expressed as the % of Plvap^+^ TECs, CD276^+^ TECs and Plvap^+^CD276^+^ TECs relative to total TEC levels for tumors with high and low levels of NP^+^ cancer cells. Statistically significant differences between high and low NP groups for every parameter is indicated where appropriate (*n* = 10; *: p < 0.05; **: p < 0.01). **c–f** The relative level of cells present in the tumor, where TAM and CAF levels are expressed relative to the total level cells in the tumor, while N-TECs are expressed as the number of CD276^+^ Plvap^+^ TECs compared to total TEC levels. The data are shown for c) group (a) which has high TAM and CAF levels and low N-TECs, **d** group (b) which has low TAM and CAF levels and high N-TECs, **e** group (c) which has high TAM and CAF and high N-TECs, and **f** group (d) which has low TAM and CAF and low N-TECs. **g** Violin plots showing the relative percentage of NP^+^ cancer cells expressed as the % of NP^+^ cancer cells as determined by ImageStreamX Mark II expressed relative to the total cancer cell population. The data are shown for groups a-d, revealing that high levels of NP^+^ cancer cells requires low TAM and CAF levels but a high ratio of N-TECs. Statistically significant differences between the different groups are indicated where appropriate (*n* = 10; *: p < 0.05; ****: p < 0.0001)

Interestingly, for NP delivery to tumor cells the level of perfusion does not seem to play a role, nor does the level of blood vessels or the maturity of the blood vessels themselves. However, while tumors with high levels of tumor cell-associated NPs were found to have low TEC levels, the ratio of TECs with upregulated expression of *CD276* and *Plvap* was found to be significantly higher (Fig. 8a, b).

These data indicate that the so-called N-TECs play a prominent role in NP delivery to the TME as well as to the tumor cells themselves. Further analysis revealed that for higher tumor cell delivery efficacy, low TAM and CAF levels combined with a high relative ratio of *CD276*^+^*Plvap*^+^ TECs could be used as potential biomarkers to examine the suitability of the tumor itself for NP delivery (Fig. 8c–g), or, in the future upon more detailed characterization of various tumor models, may serve as tools to predict NP tumor cell delivery efficacy. Taken together, these data reveal the complex multicomponent interaction of the TME and tumor-associated factors in targeting tumor cells by NPs for therapeutic or diagnostic applications.

## Discussion

The data presented in this manuscript underlie the importance of a careful characterization of tumor-specific parameters in view of predicting the ability to deliver nanosized formulations to these tumors. The data reveal that, depending on the size of the NPs, different tumor-related parameters play a role in the overall delivery efficacy of NPs to solid tumors. Starting from 40 nm diameter and larger, the results appeared to be quite similar, where a large number of parameters play an important role, being the exact composition of the TME, and specifically the TAM, CAF and TEC levels, the level of tumor perfusion and, somewhat surprisingly, the maturity level of the tumor-associated blood vessels. In line with recent reports [13], the classical view of EPR being the main contributor of NP delivery to solid tumors does not fit with the generated data. Contrary to that, more mature blood vessels with high levels of pericyte coverage promote NP delivery to solid tumors. Interestingly, irrespective of NP size, NP delivery efficacy was found to correlate to the level of *CD276* and, to a lesser extent, *Plvap* expression in TEC. These genes could therefore serve as important markers to take into account in those studies aiming to investigate NP delivery to solid tumors and could prove pivotal in trying to improve delivery efficacies in more translational settings.

The data obtained in this study reflect the situation where NPs are administered intravenously in tumor-bearing mice and have looked at which parameters mainly influence the overall delivery of NPs to solid tumors. More specifically, the aim was to evaluate whether some essential parameters could be defined that can be used to assess whether the tumor is suitable for NPs delivery or not. Overall, the delivery of NPs to solid tumors is a complex multifactorial process, where various groups have been trying to modify tumor-related parameters including TME modulation, changing particle size, transcytosis enabled tumor penetration or cell penetrating peptide modification [36]. The difficulty in interpreting the data obtained is that there is no clear optimal way forward and many, seemingly contradictory findings, have been obtained that hold great potential in their own way. One example includes the role of angiogenesis, where promotion of angiogenesis by nitric oxide-carrying micelles increased angiogenesis and resulted in improved influx of chemotherapeutics [37]. A second study used Cu-chelators to block angiogenesis and found that chemotherapy-loaded micelles carrying these chelators improved therapeutic outcome compared with chemotherapy alone [38]. It is therefore important to understand the role of NP delivery efficacy in the desired application and the type of therapy that will be applied as these, together with potential toxic side-effects, may help to make more informed decisions on which strategy to follow. Overall, more comparative studies are needed to really define which strategies are better than others in terms of delivery efficacy and safety.

The current study employed Au NPs as the model NP of choice, spanning a range from 10 to 80 nm. It is therefore important to note that various aspects such as NP shape or rigidity have not been considered in this study as that would make the entire study too big with too many parameters to consider. The results presented here should therefore be interpreted mainly in view of inorganic NPs, as for more organic NPs such as lipid-based or polymeric NPs of reduced rigidity, the ability of the NP shapes to adjust due to environmental pressures may affect how these NPs reach the tumor or tumor cells, specifically. However, the methodology presented here allows for a wide range of follow-up studies where these aspects could be considered, or where therapeutic and/or pharmacological approaches could be tested in view of their potential improvement in tumor delivery efficacy of systemically administered NPs. The data presented here also show that while clear trends can be observed and defined in view of overall tumor delivery efficacy, the results were not uniformly clear in indicating the level of NPs in the cancer cells themselves. Of interest, the ratio of *CD276*^+^*Plvap*^+^ TEC relative to total TEC levels, rather than their absolute numbers, together with low levels of TAM and CAF play an important role in promoting tumor cell-specific NP delivery. These parameters could serve as potential biomarkers to determine whether a tumor is suitable for NP delivery or not, and to help finetune where the NPs will end up inside the TME.

## Materials and methods

### Cell lines

Renca cell line was obtained from ATCC and cultured in RPMI 1640 supplemented with 10% fetal bovine serum (FBS), 1% penicillin/streptomycin, 1% non-essential amino acids and 1% sodium pyruvate (Gibco, Invitrogen, Merelbeke, Belgium). The cells were kept in a humidified 37 °C incubator with a 5% CO_2_ environment.

### Characterization of AuNPs

The core size of the AuNPs were characterized by transmission electron microscopy (TEM). Formvar film coated 400-mesh copper grids (Agar Scientific Ltd., England) were first glow-discharged to improve adsorption efficiency. Next, 10 µL of diluted NP sample (1/3 of each stock suspension) was dropped onto the grids and left to evaporate. The grids were examined using a JEM-1400 transmission electron microscope (JEOL, Japan) at accelerating voltage 80 keV. Hydrodynamic radii were measured on a PCS 100 spectrometer (Malvern, UK) at 25 °C, measuring the scattered light at a 90° angle. Samples were diluted with PBS until 0.5 mg/mL. The average value of 3 series of 10 different runs is given. Electrophoretic mobilities of the samples at identical dilutions were measured with a Zetasizer IIC instrument (Malvern, UK) at 25 °C. Nanoparticle tracking analysis was performed using a NanoSight NS300 system (Malvern, UK) using samples diluted in PBS containing 50% FBS at a final concentration of 5 µg/mL.

### Tumor model

All animal experiments and research procedures were conducted in accordance with the declaration of Helsinki and EU Directive 2010/63/EU on the protection and welfare of animals used for scientific research. These experiments were approved by the Institutional Animal Care and Research Advisory Committee (KU Leuven) (ECD number: P203/2019) and were performed in accordance with the institutional and national guidelines and regulations. Female Balb/c mice, 5 weeks old with body weights of 18–25 g, were purchased from Charles River (Wilmington, MA, US) and housed in a specific pathogen-free environment.

1 × 10^6^ Renca Firely-Luciferase and GFP-positive (Renca Luc/GFP) cells were injected subcutaneously in the right flanks of the mice to asses subcutaneous tumor growth. Tumor volumes were measured with calipers and calculated using the formula V = ∏ × ((d^2^xD)/6), where d is the minor tumor axis and D the major tumor axis.

### Tumor vascular perfusion and permeability

When the tumor sizes reached to 80 mm^3^, the tumor vascular perfusion and permeability was determined by injecting intravenous (250 ug/ml) bovine serum albumin functionalized with Alexa Fluor 555 (BSA-AF555, Thermo Fisher Scientific, Ghent, Belgium) and measured in vivo using the IVIS Spectrum (Perkin Elmer, Life Sciences, Zaventem, Belgium) during 90 min with sequential measurements every 3 min (10 s, medium binning, excitation 500 nm and emission 580 nm). Measurements occurred immediately upon intravenous administration of BSA.

### Animal experiment

Mice were injected intravenously with AuNPs (150 µg Au/mouse in 100 µL) 24 h following the perfusion/permeability assay, with sizes ranging from 10, 20, 40, 60 or 80 nm. The animals were sacrificed with Dolethal (Pentobarbital Sodico) (200 mg/ml, Vetoquinol, Aartselaar, Belgium) 72 h after the AuNPs injections. The tumor and all the major organs (heart, lungs, spleen, kidney and liver) were fixed in 4% paraformaldehyde (PFA, Klinipath, VWR, PA, USA).

### Histology, immunostainings and morphometric analysis

Mouse tissues were dehydrated after being fixed for minimum 48 h in 4% PFA at 4 °C. The tissues were after subsequently embedded in OCT compound (Sakura-Finetek, CA, USA) and frozen at -80 °C. Tissue slices of 10 µm were cut using the Cryostar NX70 (Thermo Fisher Scientific, Ghent, Belgium) and placed on glass microscope slides (VWR, PA, USA). For morphometric analyses, optical fields (40 × magnification) of the whole sections were taken by the high content screening microscope Nikon-Marzhauser Slide Express (Märzhäuser Wetzlar GmbH & Co. KG, Wetzlar, Germany) or Vectra Polaris multispectral imaging system (Perkin Elmer, Life Sciences, Zaventem, Belgium) and analysed using QuPath. The images were automatically stitched together to cover the entire slide and were saved as.OME TIFF of.qptiff file format for further analysis.

#### Hematoxylin and eosin staining for determination of tumor necrosis and tissue damage

Hematoxylin and eosin (H&E) staining was performed on 10 µm thick OCT-embedded frozen tissues obtained as described above. The tumor slides where first air-dried without dehydration for 30’ at RT and washed with 1X Phosphate buffered saline (PBS, Gibco, Thermo Fisher Scientific, Ghent, Belgium) for 5’. The slides were then stained protected from light with hematoxylin (Sigma–Aldrich, Merck, Overijse, Belgium) for 3’, washed with deionized water obtained from a Milli-Q system (MQ; Millipore, France) water for 5’, incubated for 1’ in ethanol 80% (0.15% HCl), washed for 1’ with MQ and incubated for 30’’ with ammonium-containing water, followed by washing for 5’ with MQ water and 95% ethanol for 1’. The tumor slides were incubated protected from light for 1’ in eosin (Sigma–Aldrich, Merck, Overijse, Belgium) and dehydrated after in 95% ethanol for 5’, twice in 100% ethanol for 5’ each followed by washing in Xylene (Sigma–Aldrich, Merck, Overijse, Belgium) twice for 5’ each. Finally, the samples were mounted with DPX mounting medium (Merck, Overijse, Belgium).

Tumor necrosis was then expressed as the percentage of the total tumor area as determined on H&E-stained sections. For this, the images were loaded as Brightfield (H&E) images, and a thresholder was created to classify pixels for eosin (tumor tissue) or background signal. This was followed by creating a thresholder for classifying pixels for hematoxylin signal, to discriminate healthy from necrotic tissue. The tumor area was then calculated based on the eosin stain, while the area of the healthy tissue is then calculated based on the hematoxylin stain. The relative level of necrosis is then calculated as follows: ((eosin area – hematoxylin area)/eosin area)*100 and expressed as percentage for all tissue sections and all tumors (Provide exemplary figure composed of all stages in Additional file 1.

#### Picrosirius red staining for determination of tumor extracellular matrix level

The tumor slides were air-dried without dehydration for 30’ at RT and washed with 1X PBS (Gibco, Thermo Fisher Scientific, Ghent, Belgium) for 5’. The tumor samples were stained with Picrosirius red (PSR, Abcam, Cambridge, UK) and Fastgreen (0.1 g FCF in acidified PSR solution, Sigma-Aldrich, Merck, Overijse, Belgium) for 1 h at 4 °C and rinsed twice with 0.5% acidified water followed by washing in Ethanol absolute for 30″ one time and for 5’ two times. The samples were cleaned twice in Xylene for 5’ and mounted with DPX mounting medium (Merck, Overijse, Belgium).

Tumor extracellular matrix content was expressed as the percentage of the total tumor area. For analysis in QuPath, the images were loaded as Brightfield (H-DAB) images, and the images were preprocessed and stain vectors were estimated and adjusted to represent the PSR and Fastgreen signal, respectively (see Additional file 1: Figure S3). Using the combined colours, a thresholder was created to classify pixels for tumor tissue or background signal. This was followed by creating a thresholder for classifying pixels for PSR signal, to discriminate collagen fibres from other tissue. The tumor area was then calculated based on the combined stain, while the area of the collagen is then calculated based on the PSR stain. The relative level of necrosis is then calculated as follows: (PSR area/total area)*100 and expressed as percentage for all tissue sections and all tumors.

#### Immunohistochemistry

The tumor samples were air-dried at RT for 30’ without dehydration and were washed in 1X PBS for 5’. The samples were fixed in 100% cold MeOH for 6’ at -20 °C, washed for 5’ in 1XPBS, and incubated for 15’ with Proteinase K (1:500, in 1XPBS, Promega B.V., Leiden, The Netherlands) at 37 °C. After washing the slides 5’ with 1XPBS, the slides were blocked for 1 h at RT with 1XPBS + 10% normal goat serum (NGS, 60.0 mg/mL, ThermoFischer Scientific, Ghent, Belgium) and 1%FBS and washed again twice with 1XPBS for 5’ each. The samples were blocked with Avidin (0.001%, Avidin from egg white, Sigma-Aldrich, Merck Chemicals, Overijse, Belgium) for 20’, followed by washing with 1XPBS two times for 2’ each and blocked again with Biotin (0.001%, Sigma-Aldrich, Merck, Overijse, Belgium) for 20’ and washed twice for 2’ in 1XPBS each. The samples were incubated with anti-CD31 antibody (1:25, in 1XPBS + 1%NGS, Abcam, Cambidge, UK) overnight at 4 °C.

After leaving the samples for 20’ at RT, the samples were washed twice in 1XPBS for 5’ each, blocked for 20’ with hydrogen peroxidase (3%, Alexa Fluor 594 Tyramide SuperBoost Kit, Invitrogen, Thermo Fisher Scientific, Ghent, Belgium), washed twice in 1XPBS for 5’ each and incubated with Goat anti-Rat-Biotin (1:300, in 1XPBS + 1%NGS, Jackson ImmunoResearch Europe Ltd, Ely, UK) at RT for 1 h.

The samples were washed twice in 1XPBS for 5’ each, incubated with streptavidin-HRP (1:150, in 1XPBS + 1%NGS, Invitrogen, ThermoFischer Scientific, Ghent, Belgium) for 30’ at RT, washed again two times in 1XPBS 5’ each and incubated with Alexa Fluor Tyramide 594 (1:100, Alexa Fluor 594 Tyramide SuperBoost Kit, Invitrogen, ThermoFischer Scientific, Ghent, Belgium) + hydrogen peroxidase 3% (1:100, Alexa Fluor 594 Tyramide SuperBoost Kit, Invitrogen, Thermo Fisher Scientific, Ghent, Belgium) + Tris Buffer HCl pH 7,4 (1:1) for 10’ at RT. The reaction was stopped using Stop Reagent (1:11, in 1XPBS, Alexa Fluor 594 Tyramide SuperBoost Kit, Invitrogen, ThermoFischer Scientific, Ghent, Belgium) for 2’ at RT, washed three times in 1XPBS for 5’ and incubated with anti-laminin (1:200, in 1XPBS + 1%NGS, Sigma- Aldrich, Merck Chemicals, Overijse, Belgium) or with anti-neural/glial antigen 2 (NG-2, 1:100, in 1XPBS + 1%NGS, Abcam, Cambidge, UK), overnight at 4 °C. The samples are from here on divided in two parts; samples stained with the primary anti-CD31 antibody together with the primary anti-laminin antibody, and samples stained with the primary anti-CD31 antibody together with the primary anti-NG2 antibody.

##### Anti-CD31 and anti-laminin co-staining

The samples were washed twice in 1XPBS for 5’ each, incubated with Goat Anti-Rabbit-AF488 (1:1000, in 1XPBS + 1%NGS, Invitrogen, Thermo Fisher Scientific, Ghent, Belgium) for 1 h at RT, washed twice in 1XPBS for 5’ each and incubated for 10’ at RT with Hoechst (1:1000, in 1XPBS, Invitrogen, Thermo Fisher Scientific, Ghent, Belgium). Finally, the samples were washed twice in 1XPBS for 5’ each, mounted with Fluoromont (Sigma-Aldrich, Merck Chemicals, Overijse, Belgium), air-dried for 30’ with dehydration and sealed the cover slides with transparent nail polish.

##### Anti-CD31 and anti-NG2 co-staining

The samples were washed twice in 1XPBS for 5’ each, incubated with Goat Anti-Rabbit IgG secondary antibody poly HRP (1:1, Alexa Fluor 488 Tyramide SuperBoost Kit, goat anti-rabbit IgG, Invitrogen, Thermo Fisher Scientific, Ghent, Belgium) 1 h at RT, washed twice in 1XPBS for 5’ each, incubated with Alexa Fluor Tyramide 488 (1:100, Alexa Fluor 488 Tyramide SuperBoost Kit, goat anti-rabbit IgG, Invitrogen, Thermo Fisher Scientific, Ghent, Belgium) + hydrogen peroxidase 3% (1:100, Alexa Fluor 488 Tyramide SuperBoost Kit, goat anti-rabbit IgG, Invitrogen, Thermo Fisher Scientific, Ghent, Belgium) + Tris Buffer HCl pH 7,4 (1:1) for 10’ at RT, incubated after for 2’ at RT with Stop Reagent (1:11, in 1XPBS, Alexa Fluor 488 Tyramide SuperBoost Kit, goat anti-rabbit IgG, Invitrogen, Thermo Fisher Scientific, Ghent, Belgium), washed three times in 1XPBS for 5’ each and incubated for 10’ at RT with Hoechst (1:1000, in 1XPBS, Invitrogen, Thermo Fisher Scientific, Ghent, Belgium). Finally, the samples were washed twice in 1XPBS for 5’ each, mounted with Fluoromont (Sigma-Aldrich, Merck Chemicals, Overijse, Belgium), air-dried for 30’ with dehydration and sealed the cover slides with transparent nail polish.

Tumor vessel area was analysed by immunostaining CD31, which is a marker for endothelial cells, where the total CD31^+^ area was expressed as the percentage of the total tumor area. For analysis in QuPath, the entire image was annotated as a field (tumor tissue), where the number of cells were calculated using “cell detection” based on DAPI signal. Positive cell detection was then performed by setting threshold for the AF594 signal. The total number of endothelial cells then were determined as well as the total area of CD31^+^ positive cells compared to other cells. The relative level of CD31^+^ vessel density is then calculated as follows: ((CD31 area)/total area)*100 and expressed as percentage for all tissue sections and all tumors (please see Additional file 1: Figures S6, S7 for examples).

Tumor vessel maturation was assessed by the immunostaining for NG-2, which is a pericyte marker together with immunostaining for CD31. For analysis in QuPath, the entire image was annotated as a field (tumor tissue), where the number of cells were calculated using “cell detection” based on DAPI signal. Positive cell detection was then performed by setting threshold for the AF594 signal (CD31 positive) or AF488 signal (NG2 positive). For analysis, the CD31^+^ cells and NG2^+^ cells were artificially dilated twofold and any enlarged cell (typically the size of at least 2 cells), comprising both CD31 and NG2 signal is determined as a mature vessel. The total number of mature endothelial cells then were determined as well as the total area of NG2^+^CD31^+^ double positive cells compared to total number of CD31^+^ endothelial cells. The relative level of NG2^+^CD31^+^ vessel density is then calculated as follows: ((NG2^+^CD31^+^ area)/CD31^+^ area)*100 and expressed as percentage for all tissue sections and all tumors (Please see Supplementary Figure S7 as an example).

In addition, basement membrane deposition was assessed by immunostaining for laminin. The number of Laminin^+^ vessels was defined and is expressed as percentage total vessel area. For analysis in QuPath, this occurred similarly as for NG2 analysis, where here NG2 was replaced by laminin. The total number of mature endothelial cells then were determined as well as the total area of Lam^+^CD31^+^ double positive cells compared to total number of CD31^+^ endothelial cells. The relative level of Lam^+^CD31^+^ vessel density is then calculated as follows: ((Lam^+^CD31^+^ area)/CD31^+^ area)*100 and expressed as percentage for all tissue sections and all tumors (Please see Supplementary Figure S6 as an example).

### RNAscope analysis

RNA in situ hybridization for mouse *Plvap* (440,221-C1), mouse *CD276* (590,091-C3) and mouse *CD31* (471,481-C2) was performed according to the manufacturer’s instructions (Advanced Cell Diagnostics). Briefly, a total of 10 sections were selected, 5 sections of those tissues with the highest overall level of tumor-associated NPs and 5 sections of tumors with the overall lowest level of tumor-associated NPs. Of the tissue samples, 10 μm paraformaldehyde-fixed, OCT-embedded frozen intestinal tumour sections were pretreated with heat in the retrieval reagent and protease III before hybridization with the target oligonucleotide probes. Preamplifier, amplifier and alkaline-phosphatase-labelled oligonucleotides were then hybridized sequentially. HRP signal was developed using Opal520 (Akoya Biosceinces, FP1487001KT) for the CD31 probe, Opal570 (Akoya Biosceinces, FP1488001KT) for the *Plvap* probe and Opal620 (Akoya Biosceinces, FP1495001KT) for the *CD276* probe. Quality control was performed to assess RNA integrity with probes specific to ubiquitously expressed household genes PolR2A RNA (320881-C1), PPIB RNA (320881-C2), UBC RNA (320881-C3) and for background staining with a probe specific to bacterial dapB RNA (320871). Samples were counterstained with DAPI nuclear counterstain and imaged using the Vectra Polaris multispectral imaging system (Perkin Elmer, Life Sciences, Zaventem, Belgium) and analysed using QuPath. Specific fluorescent signal for *CD31*, *Plvap* and *CD276* was identified as green, red and far-red punctate dots, respectively. For analysis, *CD31*^+^ cells were identified as cells with green positive dots and in these cells, the presence of *CD276* and *Plvap* (single or both) in these cells was determined and expressed relative to the total amount of *CD31*^+^ cells.

### Single cell analysis of NP uptake by image-based cytometry

The tumor samples were dissociated into single cells using GentleMACS tissue dissociator and its kit (Miltenyi Biotec, Gladbach, Germany). The tumor samples were cut into small pieces, transferred in gentleMACS C-tubes containing RPMI/DMEM media and kit enzymes (tumor dissociation kit, Miltenyi Biotec, Gladbach, Germany) and were broken down using the gentleMACS. After the tumors were processed, the gentleMACS C-tubes were centrifuged 30’ on 1,5 rpm, the content of the gentleMACS C-tube was passed first through 70 um, followed by 40 um strainer and was centrifuged for 7’ on 300 g. The pellet was lysed using RBC lysis buffer for exactly 2’, centrifuged for 5’ on 300 g and resuspended in 1 mL media, which was after added slowly on top of 1 mL FBS to form a layer of media on top of the FBS. After centrifugation for 5’ on 100 g, the single tumor cells inside the media sank to the bottom of the FBS and the tumor cells were separated from the debris. The supernatant was removed, the cells were washed with 1XPBS, centrifuged for 5’ on 1.4 rpm and were incubated with Fc Blocker (1:100, in 1XPBS + 1%FBS, Thermo Fisher Scientific, Ghent, Belgium) for 30’ on ice. The cells were washed with 1XPBS + 1%FBS, centrifuged for 5’ on 1.4 rpm and incubated with two different antibody cocktails for 1 h on ice, protected from light, where all the antibodies were diluted in 1XPS + 1%FBS. The following antibodies were used in the first cocktail; anti-CD45 FITC (1:100, Thermo Fisher Scientific, Ghent, Belgium), anti-90.2 APC (2:100, Thermo Fisher Scientific, Ghent, Belgium), anti-CD3 AF610 (2:100, Thermo Fisher Scientific, Ghent, Belgium) and anti-CD144 PE (2:100, Thermo Fisher Scientific, Ghent, Belgium), and the following two antibodies in the second cocktail; anti-CD24 APC (3:100, Thermo Fisher Scientific, Ghent, Belgium) and anti-F4/80 FITC (3:100, Bio-Rad Laboratories, Temse, Belgium). The single cells were washed with 1XPBS + 1%FBS, resuspended in 1XPBS and transported in eppendorfs to the image-based cytometer Imagestream Mark II Imaging flow cytometer (Merck, Overijse, Belgium). Measurements were done by acquiring approximately 1 × 10^5^ single cells per sample. The images were acquired using the 60 × objective with the darkfield (780 nm laser) at 1 mW in order to reduce scatter light, while enabling darkfield-based detection of AuNPs inside cells. For the first cocktail, laser intensities were set at 1.00mW (488 nm), 20.00 mW (561 nm) and 50.00 mW (642 nm) and for the second cocktail, these were set at 1.00 mW (488 nm) and 100 mW (642 nm).

For analysis, iDEAS software (Amnis Corporation, USA) was used, followed by FCS Express 7.0 for visualization. First, focused and single cell were selected and gated, after which cell selections were gated based on the different markers used: tumor cells were defined as CD45-CD24 + , tumor-associated macrophages as F4/80 + , leukocytes as CD45 + , endothelial cells as CD144 + and cancer associated fibroblasts as CD45-CD90.2 + . For every cell type, darkfield images were taken and signal obtained in the darkfield channel were analysed for the different cell types, using bright intensity projection. Please see Supplementary Figures S4 and S5 for the gating strategy.

### Inductively coupled mass spectrometry

#### Instrumentation

(Ultra-)trace element determination of Au was carried out using an Agilent 8800 ICP-MS/MS instrument (ICP-QQQ, Agilent Technologies, Japan). The sample introduction system comprises a concentric nebulizer (400 µL min^−1^) mounted onto a Peltier-cooled (2 °C) Scott-type spray chamber. This instrument is equipped with a tandem mass spectrometry configuration consisting of two quadrupole units (Q1 and Q2) and a collision/reaction cell (CRC) located in-between both quadrupole mass filters (Q1-CRC-Q2). All measurements were performed in MS/MS mode (on-mass approach) with the collision/reaction cell (CRC) operated in “vented” (no gas) mode.

#### Reagents and standards

For ICP-MS/MS analysis, only high-purity reagents were used. Ultra-pure water (resistivity 18.2 MΩ cm) was obtained from a Milli-Q Element water purification system (Millipore, France). Pro-analysis purity level 14 M HNO_3_ (Chem-Lab, Belgium) further purified by sub-boiling distillation and ultra-pure 9.8 M H_2_O_2_ (Sigma Aldrich, Belgium) were used for sample digestion. Appropriate dilutions of 1 g L^−1^ single element standard solutions of Au and Tl (Inorganic Ventures, USA) were used for method development, optimization, and calibration purposes. For quantitative element determination of Au, external calibration was relied on as calibration approach (0, 0.1, 0.25, 0.5, 1.0 and 2.5 µg L^−1^ Au), with Tl (2 µg L^−1^) as internal standard.

#### Samples and sample preparation

The samples were digested via acid digestion in Teflon Savillex® beakers, which had been pre-cleaned with HNO_3_ and HCl and subsequently rinsed with Milli-Q water. A mixture of 1 mL of 14 M HNO_3_ and 0.5 mL of 9.8 M H_2_O_2_ was added to each sample (mass ranging between 1 and 770 mg) and the procedure was completed after heating at 110 °C on a hot plate for approximately 18 h. Prior to ICP-MS/MS analysis, the digests were appropriately diluted (between 10- and 2000-fold dilution) with Milli-Q water. To avoid contamination, only metal-free tubes were used for standard and sample preparation (15 mL polypropylene centrifuge tubes, VWR, Belgium). Tl was added to all samples and standards to correct for instrument instability, signal drift and matrix effects.

### NP biodistribution analysis per parameter

The effect of the different parameters measured (NP diameter, tumor size, ECM density, necrotic area, vessel perfusion, vessel permeability, blood vessel area, blood vessel maturity, blood vessel-ECM, number of TAMs, number of CAFs, number of endothelial cells, number of lymphocytes) on NP distribution (efficacy of NP accumulation in total tumor area and efficacy of NP accumulation in tumor cells) is calculated using Uniform Manifold Approximation and Projection (UMAP) based dimensionality reductions (FlowExpress 7.0) to assess which parameters caused the biggest influence on NP tumor accumulation.

### Blood biochemistry

Upon isolation of the tumors and main organs for analysis, blood samples were also collected of control mice bearing Renca tumors but without any AuNPs or mice Balb/c mice with Renca tumors having received a bolus of AuNPs. Blood samples were collected retroorbitally following animal sacrifice (200 µl/animal), and samples were c7ollected and centrifuged in heparin-containing tubes to separate plasma from serum (15 min at 3500 rpm). Next, 75 µl serum was added on analysis discs (Samsung Comprehensive test 16 V) enabling analysis of 16 different markers using the Samsung PT10V chemistry analyzer (SCIL Animal care company GmbH, Viernheim, Germany). The following markers were analyzed: glucose, urea, creatinine, urea/creatinine ratio, phosphates, calcium, total protein, albumin, globulin, albumin-globulin ratio, alanine aminotransferase, alkaline phosphatases, bilirubin, cholesterol, triglycerides and amylase.

### Statistical analysis

All statistical analyses were performed using GraphPad 9.0 statistical analysis software. To determine significant differences between groups, 2-way ANOVA tests were performed with Tukey post-hoc test, unless otherwise indicated in the corresponding text. The levels of significance and number of independent repeats are indicated with every data point given.

## Supplementary Information

**Additional file 1: Figure S1.** Overview of necrosis level detection in H&E-stained tissue sections using QuPath software. a) A representative image of an H&E stained tumor tissue section will be loaded as H&E brightfield image in QuPath software. Classifiers are then generated for eosin and hematoxylin stains, respectively. b) A threshold is selected that will indicate eosin-positive tissue (highlighted in red) from background signal (white gaps delineated with yellow lines). c) A threshold is then set up for hematoxylin signal (dark yellow), which will delineate the more dense, cell-loaded sections of the tumor. The original ‘gaps’ delineated using the eosin stain (white gaps with yellow demarcations) are retained. The size of the total tissue section (the red selection in b) is calculated as well as the size of the healthy tissue (the dark yellow selection in c). The level of necrosis is then calculated as: (total area – healthy area)/total area * 100 and expressed as % necrosis. **Figure S2.** Overview of ECM level in PSR and FastGreen-stained tissue sections using QuPath software. a) A representative image of a PSR and FastGreen-stained tumor tissue section will be loaded as an H-DAB brightfield image in QuPath software. As the associated classifiers are then generated for H-DAB, these need to be adjusted and the images are preprocessed where stain vectors are automatically estimated and then adjusted to represent the PSR and Fastgreen signal, respectively (bottom figures). b) A threshold is selected that will indicate Fastgreen-positive tissue (highlighted in red) from background signal (white gaps). c) A threshold is then set up for PSR signal (yellow), which will delineate the more dense, cell-loaded sections of the tumor. The original ‘gaps’ delineated using the eosin stain (white gaps with red demarcations) are retained. The size of the total tissue section (the red selection in b) is calculated as well as the size of the collagen-positive ECM tissue (the yellow selection in c). The level of ECM is then calculated as: ECM area/total area * 100 and expressed as % ECM. **Figure S3.** Gating strategy for TAM and CC populations by image-based flow cytometry of single cell suspensions. a) A representative image of collected cells, showing the entire population of cells, where cells in focus are selected upon selecting the gradient RMS and visual inspection of selected cells. b) Focused cells are then plotted in function of the aspect ratio and total area of the cells, where viable single cells (no debris or doublets) are selected as having an aspect ratio of > 0.8 and an area of 50 -250 px. c) Gated viable and single cells are then plotted for the level of antibody stained CD24 (cancer cell marker) and F4/80 (TAM marker), and the gates were selected to indicate the pure populations. Visual inspection of the cells displays the corresponding single colour of the appropriate marker and by darkfield imaging (Ch06), the presence or absence of Au NPs inside the cell can be detected. **Figure S4.** Gating strategy for TIL, CAF and TEC populations by image-based flow cytometry of single cell suspensions. a) A representative image of collected cells, showing the entire population of cells, where cells in focus are selected upon selecting the gradient RMS and visual inspection of selected cells. b) Focused cells are then plotted in function of the aspect ratio and total area of the cells, where viable single cells (no debris or doublets) are selected as having an aspect ratio of > 0.8 and an area of 50-250 px. c) Gated viable and single cells are then plotted for the level of antibody stained CD45 (immune cells) and CD45+CD3+ (TIL marker) and the gates were selected to indicate the pure populations. d) From the CD45- population, cells were plotted for presence of antibody stained CD144 (TEC marker) and CD90.2 (CAF marker) and the gates were selected to indicate the pure populations. **Figure S5.** Overview of analysis strategy for detection of laminin-covered tumor-associated blood vessels in antibody-stained tissue sections using QuPath software. a) A representative image of a tissue section stained for CD31 and laminin and counterstained with DAPI nuclear stain. b) Using DAPI nuclear stain, a threshold was selected that generated a positive selection (red selection markers) of individual cells. As DAPI only provides a nuclear stain, the area was expanded by 5 µm around all edges. c) Using the cell selection, a first classifier was generated, where cells positive for CD31 in the extended cytoplasm were selected (positive cells: red marking; negative cells: green marking). d) Using the first classifier, a second classifier can be generated, where CD31+ cells were evaluated for the presence of laminin. The total number of CD31+ cells, laminin+ cells, or CD31+ cells located next to laminin+ cells were then determined. The level of laminin-covered CD31+ cells in function of total CD31+ cell level is then calculated and expressed as % laminin. Scale bars: 50 µm. **Figure S6.** Overview of analysis strategy for detection of NG2-covered tumor-associated blood vessels in antibody-stained tissue sections using QuPath software. a) A representative image of a tissue section stained for CD31 and NG2 and counterstained with DAPI nuclear stain. b) Using DAPI nuclear stain, a threshold was selected that generated a positive selection (red selection markers) of individual cells. As DAPI only provides a nuclear stain, the area was expanded by 5 µm around all edges. c) Using the cell selection, a first classifier was generated, where cells positive for CD31 in the extended cytoplasm were selected (positive cells: red marking; negative cells: green marking). d) Using the first classifier, a second classifier can be generated, where CD31+ cells were evaluated for the presence of NG2. The total number of CD31+ cells, NG2+ cells, or CD31+ NG2+ cells were then determined. The level of NG2-covered CD31+ cells in function of total CD31+ cell level is then calculated and expressed as % NG2. **Figure S7.** Blood biochemistry results indicate no toxicity of Au NPs. Histograms showing blood biochemistry results of Renca-bearing control mice (light grey bars) or Renca-bearing mice exposed to a) Au10 NPs, b) Au20 NPs, c) Au40 NP, d) Au60 NPs, e) Au80 NPs. All data are expressed as mean + SEM (n = 6). The following markers are studied: glucose (GLU), blood urea nitrogen (BUN), creatinine (CREA), ratio of urea over creatinine (B/C), phosphates (PHOS), calcium (CA), total protein (TP), albumin (ALB), globuline (GLOB), ratio of albumin over globuline (A/G), alanine aminotransferase (ALT), alkaline phosphatase (ALP), gamma-glutamyl transpeptidase (GGT), total bilirubin (TBIL), cholesterol (CHOL), triglycerides (LIPA), alpha-amylase (AMY). **Figure S8.** Macroscopic organ examinations do not reveal toxicity by Au NPs. Representative H&E stained micrographs of tissue slices obtained from the heart (left column), lung (second column), spleen (middle column), kidney (4th column), liver (right column). Images are shown for Renca-bearing animals that were administered PBS (top row), Au10 NPs (2nd row), Au20 NPs (3rd rown), Au40 NPs (4th row), Au60 NPs (5th row), Au80 NPs (bottom row). **Figure S9.** UMAP plots for each and every tumor-associated parameter determined (as displayed in Figure 4) as a function of UMAP coordinates. For analysis, all data points for each and every animal were combined for animals receiving Au10 NPs. For every parameter, the values were first rescaled to a linear 0-1 scale with 0 being the lowest value for that parameter across all animals and 1 being the highest value for that parameter across all animals. The dots were colour-coded based on the upper left plot (the total tumor NP uptake level), where the 25% of animals with highest NP tumor levels were coloured red, the 25% of animals with lowest NP tumor levels were coloured blue and the remaining animals with medium NP levels were coloured green. To determine whether a particular parameter promotes or inhibits NP delivery efficacy, the red and blue groups should be separated on the Y-axis for the parameter. The parameters with most distinction were tumor necrosis and ECM. **Figure S10.** UMAP plots for each and every tumor-associated parameter determined (as displayed in Figure 4) as a function of UMAP coordinates. For analysis, all data points for each and every animal were combined for animals receiving Au20 NPs. For every parameter, the values were first rescaled to a linear 0-1 scale with 0 being the lowest value for that parameter across all animals and 1 being the highest value for that parameter across all animals. The dots were colour-coded based on the upper left plot (the total tumor NP uptake level), where the 25% of animals with highest NP tumor levels were coloured red, the 25% of animals with lowest NP tumor levels were coloured blue and the remaining animals with medium NP levels were coloured green. To determine whether a particular parameter promotes or inhibits NP delivery efficacy, the red and blue groups should be separated on the Y-axis for the parameter. The parameters with most distinction were tumor size, TEC level and perfusion. **Figure S11.** UMAP plots for each and every tumor-associated parameter determined (as displayed in Figure 4) as a function of UMAP coordinates. For analysis, all data points for each and every animal were combined for animals receiving Au40 NPs. For every parameter, the values were first rescaled to a linear 0-1 scale with 0 being the lowest value for that parameter across all animals and 1 being the highest value for that parameter across all animals. The dots were colour-coded based on the upper left plot (the total tumor NP uptake level), where the 25% of animals with highest NP tumor levels were coloured red, the 25% of animals with lowest NP tumor levels were coloured blue and the remaining animals with medium NP levels were coloured green. To determine whether a particular parameter promotes or inhibits NP delivery efficacy, the red and blue groups should be separated on the Y-axis for the parameter. The parameters with most distinction were tumor necrosis, tumor ECM, TAM, CAF, TEC, CC levels, vessel area, NG2+ vessel levels and perfusion. **Figure S12.** UMAP plots for each and every tumor-associated parameter determined (as displayed in Figure 4) as a function of UMAP coordinates. For analysis, all data points for each and every animal were combined for animals receiving Au60 NPs. For every parameter, the values were first rescaled to a linear 0-1 scale with 0 being the lowest value for that parameter across all animals and 1 being the highest value for that parameter across all animals. The dots were colour-coded based on the upper left plot (the total tumor NP uptake level), where the 25% of animals with highest NP tumor levels were coloured red, the 25% of animals with lowest NP tumor levels were coloured blue and the remaining animals with medium NP levels were coloured green. To determine whether a particular parameter promotes or inhibits NP delivery efficacy, the red and blue groups should be separated on the Y-axis for the parameter. The parameters with most distinction were tumor necrosis, tumor ECM, TAM, CAF, TEC levels, vessel area, NG2+ vessel levels and perfusion. **Figure S13.** UMAP plots for each and every tumor-associated parameter determined (as displayed in Figure 4) as a function of UMAP coordinates. For analysis, all data points for each and every animal were combined for animals receiving Au80 NPs. For every parameter, the values were first rescaled to a linear 0-1 scale with 0 being the lowest value for that parameter across all animals and 1 being the highest value for that parameter across all animals. The dots were colour-coded based on the upper left plot (the total tumor NP uptake level), where the 25% of animals with highest NP tumor levels were coloured red, the 25% of animals with lowest NP tumor levels were coloured blue and the remaining animals with medium NP levels were coloured green. To determine whether a particular parameter promotes or inhibits NP delivery efficacy, the red and blue groups should be separated on the Y-axis for the parameter. The parameters with most distinction were tumor necrosis, tumor ECM, TAM, CAF, TEC levels, vessel area, NG2+ vessel levels and perfusion. **Figure S14.** Gating strategy for NP-containing cells. The selection of CC and TAM is illustrated in Supporting Figure S4, for TIL, CAF and TEC, this is illustrated in Supporting Figure S5. Upon selection of the gated cells, a new histogram is created that plots the Bright Detail Intensity of the masked cellular region in Ch06 (dark field channel). Only bright dots, indicating the presence of NPs, will generate positive contrast in the images. For the smaller NPs, this was validated using the corresponding fluorescence signal of the AF647-coupled NPs and overlapping spots in Ch05 and Ch06 of in vitro labeled cells. The threshold for detection was determined using control animals bearing Renca tumors that had never been given any NPs. The number of cells positive for NPs in the corresponding cell types can then be determined and expressed as the % of NP+ cells versus the total number of that particular cell type. **Figure S15.** UMAP plots for each and every tumor-associated parameter determined (as displayed in Figure 4) as a function of UMAP coordinates. For analysis, all data points for each and every animal were combined for animals receiving Au10 NPs. For every parameter, the values were first rescaled to a linear 0-1 scale with 0 being the lowest value for that parameter across all animals and 1 being the highest value for that parameter across all animals. The dots were colour-coded based on the level of NP+ cancer cells, where the 25% of animals with highest NP+ cancer cells were coloured red, the 25% of animals with lowest NP+ cancer cells were coloured blue and the remaining animals with medium NP levels were coloured green. To determine whether a particular parameter promotes or inhibits NP delivery efficacy, the red and blue groups should be separated on the Y-axis for the parameter. **Figure S16.** UMAP plots for each and every tumor-associated parameter determined (as displayed in Figure 4) as a function of UMAP coordinates. For analysis, all data points for each and every animal were combined for animals receiving Au20 NPs. For every parameter, the values were first rescaled to a linear 0-1 scale with 0 being the lowest value for that parameter across all animals and 1 being the highest value for that parameter across all animals. The dots were colour-coded based on the level of NP+ cancer cells, where the 25% of animals with highest NP+ cancer cells were coloured red, the 25% of animals with lowest NP+ cancer cells were coloured blue and the remaining animals with medium NP levels were coloured green. To determine whether a particular parameter promotes or inhibits NP delivery efficacy, the red and blue groups should be separated on the Y-axis for the parameter. **Figure S17.** UMAP plots for each and every tumor-associated parameter determined (as displayed in Figure 4) as a function of UMAP coordinates. For analysis, all data points for each and every animal were combined for animals receiving Au40 NPs. For every parameter, the values were first rescaled to a linear 0-1 scale with 0 being the lowest value for that parameter across all animals and 1 being the highest value for that parameter across all animals. The dots were colour-coded based on the level of NP+ cancer cells, where the 25% of animals with highest NP+ cancer cells were coloured red, the 25% of animals with lowest NP+ cancer cells were coloured blue and the remaining animals with medium NP levels were coloured green. To determine whether a particular parameter promotes or inhibits NP delivery efficacy, the red and blue groups should be separated on the Y-axis for the parameter. The most distinct parameters were TAM, CAF and TEC levels. **Figure S18.** UMAP plots for each and every tumor-associated parameter determined (as displayed in Figure 4) as a function of UMAP coordinates. For analysis, all data points for each and every animal were combined for animals receiving Au60 NPs. For every parameter, the values were first rescaled to a linear 0-1 scale with 0 being the lowest value for that parameter across all animals and 1 being the highest value for that parameter across all animals. The dots were colour-coded based on the level of NP+ cancer cells, where the 25% of animals with highest NP+ cancer cells were coloured red, the 25% of animals with lowest NP+ cancer cells were coloured blue and the remaining animals with medium NP levels were coloured green. To determine whether a particular parameter promotes or inhibits NP delivery efficacy, the red and blue groups should be separated on the Y-axis for the parameter. The most distinct parameters were TAM, CAF and TEC levels. **Figure S19.** UMAP plots for each and every tumor-associated parameter determined (as displayed in Figure 4) as a function of UMAP coordinates. For analysis, all data points for each and every animal were combined for animals receiving Au40 NPs. For every parameter, the values were first rescaled to a linear 0-1 scale with 0 being the lowest value for that parameter across all animals and 1 being the highest value for that parameter across all animals. The dots were colour-coded based on the level of NP+ cancer cells, where the 25% of animals with highest NP+ cancer cells were coloured red, the 25% of animals with lowest NP+ cancer cells were coloured blue and the remaining animals with medium NP levels were coloured green. To determine whether a particular parameter promotes or inhibits NP delivery efficacy, the red and blue groups should be separated on the Y-axis for the parameter. The most distinct parameters were TAM, CAF and TEC levels.

## Data Availability

All data needed to evaluate the conclusions in the paper are present in the paper and/or the Supplementary Materials. The raw data on tumor characterization and NM delivery efficacy can be provided by the corresponding author pending scientific review and a completed material transfer agreement. Requests for the raw numerical data should be submitted to: s.soenen@kuleuven.be.
