# Supplementary material for: Gold nanoparticle delivery to solid tumors: a multiparametric study on particle size and the tumor microenvironment
Source: J Nanobiotechnology. 2022 Dec 9;20:518. doi: 10.1186/s12951-022-01727-9 (PMC9733103; doi:10.1186/s12951-022-01727-9)
Supplement: Supplementary file 1 — Additional file 1: Figure S1. Overview of necrosis level detection in H&E-stained tissue sections using QuPath software. a) A representative image of an H&E stained tumor tissue section will be loaded as H&E brightfield image in QuPath software. Classifiers are then generated for eosin and hematoxylin stains, respectively. b) A threshold is selected that will indicate eosin-positive tissue (highlighted in red) from background signal (white gaps delineated with yellow lines). c) A threshold is then set up for hematoxylin signal (dark yellow), which will delineate the more dense, cell-loaded sections of the tumor. The original ‘gaps’ delineated using the eosin stain (white gaps with yellow demarcations) are retained. The size of the total tissue section (the red selection in b) is calculated as well as the size of the healthy tissue (the dark yellow selection in c). The level of necrosis is then calculated as: (total area – healthy area)/total area * 100 and expressed as % necrosis. Figure S2. Overview of ECM level in PSR and FastGreen-stained tissue sections using QuPath software. a) A representative image of a PSR and FastGreen-stained tumor tissue section will be loaded as an H-DAB brightfield image in QuPath software. As the associated classifiers are then generated for H-DAB, these need to be adjusted and the images are preprocessed where stain vectors are automatically estimated and then adjusted to represent the PSR and Fastgreen signal, respectively (bottom figures). b) A threshold is selected that will indicate Fastgreen-positive tissue (highlighted in red) from background signal (white gaps). c) A threshold is then set up for PSR signal (yellow), which will delineate the more dense, cell-loaded sections of the tumor. The original ‘gaps’ delineated using the eosin stain (white gaps with red demarcations) are retained. The size of the total tissue section (the red selection in b) is calculated as well as the size of the collagen-positive ECM tissue (the yellow [file 12951_2022_1727_MOESM1_ESM.docx]

**Gold nanoparticle delivery to solid tumors: a multiparametric study on particle size and the tumor microenvironment.**

Mukaddes Izci^1^, Christy Maksoudian^1^, Filipa Gonçalves^1^, Lucia Aversa^1^, Robbe Salembier^1^, Ara Sargsian^2^, Irati Pérez Gilabert^1^, Tianjiao Chu^1^, Carla Rios Luci^1^, Eduardo Bolea-Fernandez^3^, David Nittner^4^, Frank Vanhaecke^3^, Bella B. Manshian^2,5^, Stefaan J. Soenen^1,5,*^

^1^NanoHealth and Optical Imaging Group, Department of Imaging and Pathology, KU Leuven Herestraat 49, B3000 Leuven, Belgium

^2^Translational Cell and Tissue Research Unit, Department of Imaging and Pathology, KU Leuven Herestraat 49, B3000 Leuven, Belgium

^3^Atomic & Mass Spectrometry – A&MS research group, Department of Chemistry, Ghent University, Campus Sterre, Krijgslaan 281-S12, 9000 Ghent, Belgium

^4^Laboratory for Molecular Cancer Biology, VIB-KU Leuven, Herestraat 49, B3000 Leuven, Belgium

^5^Leuven Cancer Research Institute, Faculty of Medical Sciences, KU Leuven, Herestraat 49, B3000 Leuven, Belgium

* Corresponding author: email: [s.soenen@kuleuven.be](mailto:s.soenen@kuleuven.be)

Tel: +32 16 330034

Supplementary information


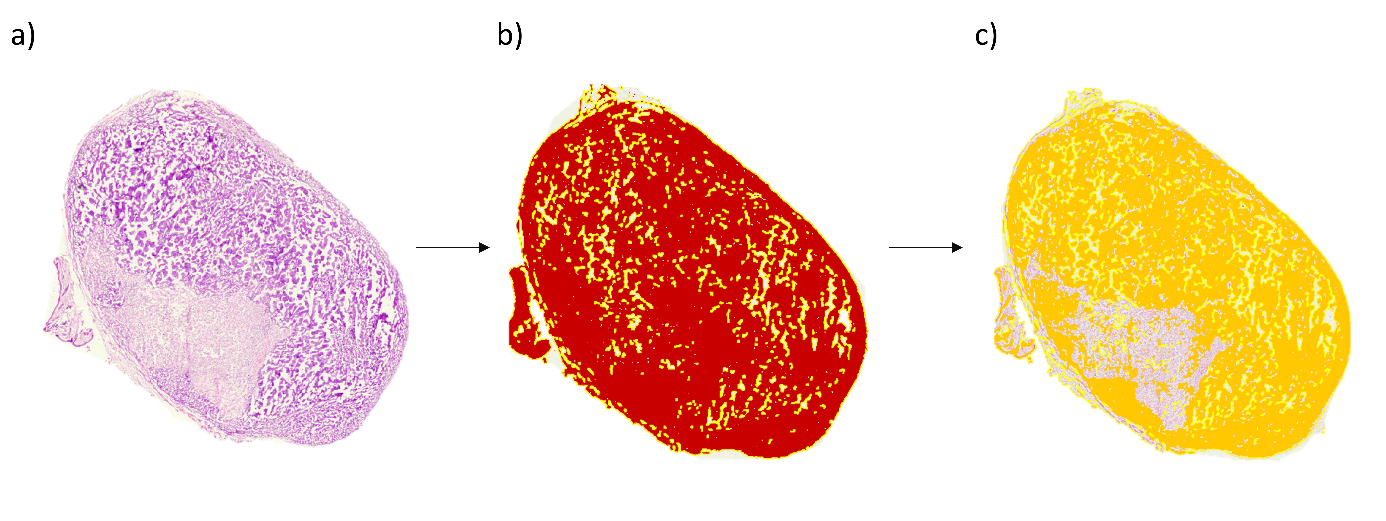


**Supplementary Figure S1. Overview of necrosis level detection in H&E-stained tissue sections using QuPath software.** **a)** A representative image of an H&E stained tumor tissue section will be loaded as H&E brightfield image in QuPath software. Classifiers are then generated for eosin and hematoxylin stains, respectively. **b)** A threshold is selected that will indicate eosin-positive tissue (highlighted in red) from background signal (white gaps delineated with yellow lines). **c)** A threshold is then set up for hematoxylin signal (dark yellow), which will delineate the more dense, cell-loaded sections of the tumor. The original ‘gaps’ delineated using the eosin stain (white gaps with yellow demarcations) are retained. The size of the total tissue section (the red selection in b) is calculated as well as the size of the healthy tissue (the dark yellow selection in c). The level of necrosis is then calculated as: (total area – healthy area)/total area * 100 and expressed as % necrosis.


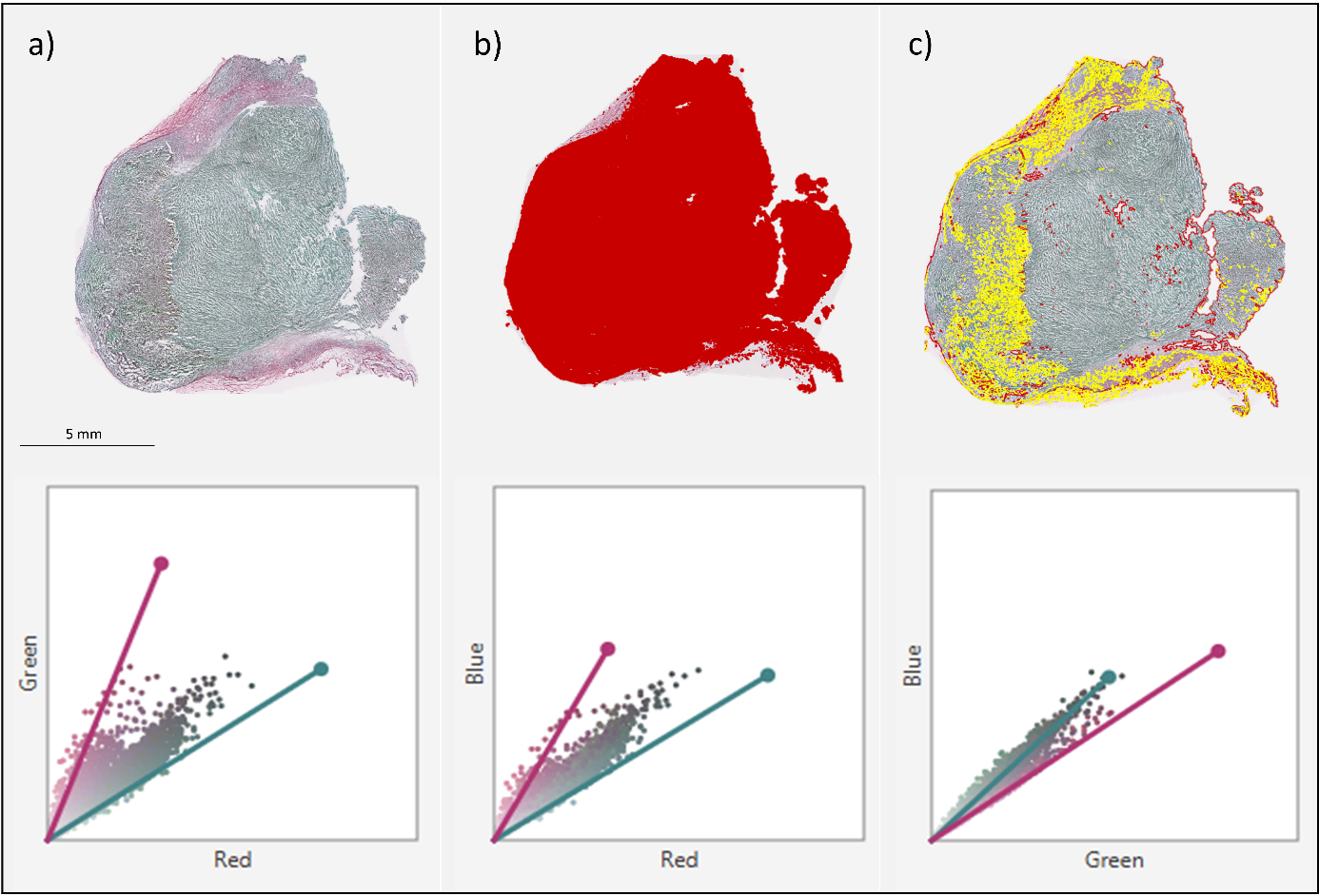


**Supplementary Figure S2. Overview of ECM level in PSR and FastGreen-stained tissue sections using QuPath software.** **a)** A representative image of a PSR and FastGreen-stained tumor tissue section will be loaded as an H-DAB brightfield image in QuPath software. As the associated classifiers are then generated for H-DAB, these need to be adjusted and the images are preprocessed where stain vectors are automatically estimated and then adjusted to represent the PSR and Fastgreen signal, respectively (bottom figures). **b)** A threshold is selected that will indicate Fastgreen-positive tissue (highlighted in red) from background signal (white gaps). **c)** A threshold is then set up for PSR signal (yellow), which will delineate the more dense, cell-loaded sections of the tumor. The original ‘gaps’ delineated using the eosin stain (white gaps with red demarcations) are retained. The size of the total tissue section (the red selection in b) is calculated as well as the size of the collagen-positive ECM tissue (the yellow selection in c). The level of ECM is then calculated as: ECM area/total area * 100 and expressed as % ECM.


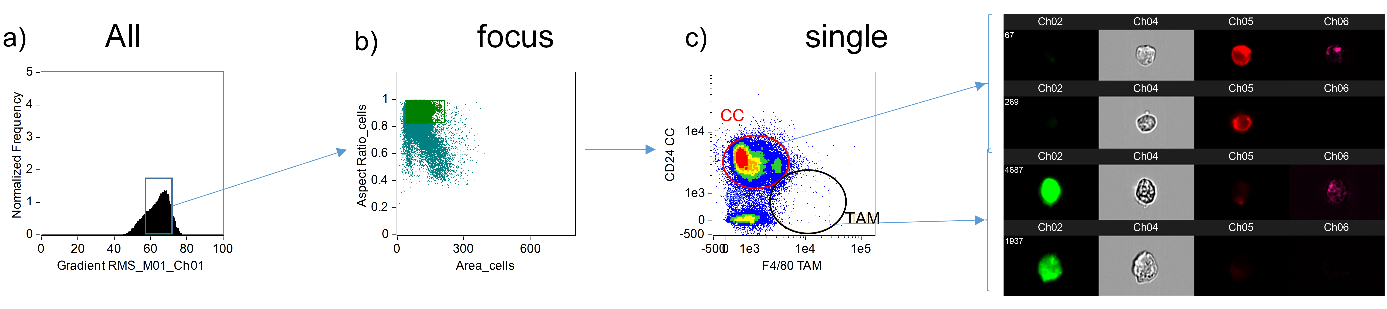


**Supplementary Figure S3. Gating strategy for TAM and CC populations by image-based flow cytometry of single cell suspensions. a)** A representative image of collected cells, showing the entire population of cells, where cells in focus are selected upon selecting the gradient RMS and visual inspection of selected cells. **b)** Focused cells are then plotted in function of the aspect ratio and total area of the cells, where viable single cells (no debris or doublets) are selected as having an aspect ratio of > 0.8 and an area of 50 -250 px. **c)** Gated viable and single cells are then plotted for the level of antibody stained CD24 (cancer cell marker) and F4/80 (TAM marker), and the gates were selected to indicate the pure populations. Visual inspection of the cells displays the corresponding single colour of the appropriate marker and by darkfield imaging (Ch06), the presence or absence of Au NPs inside the cell can be detected.


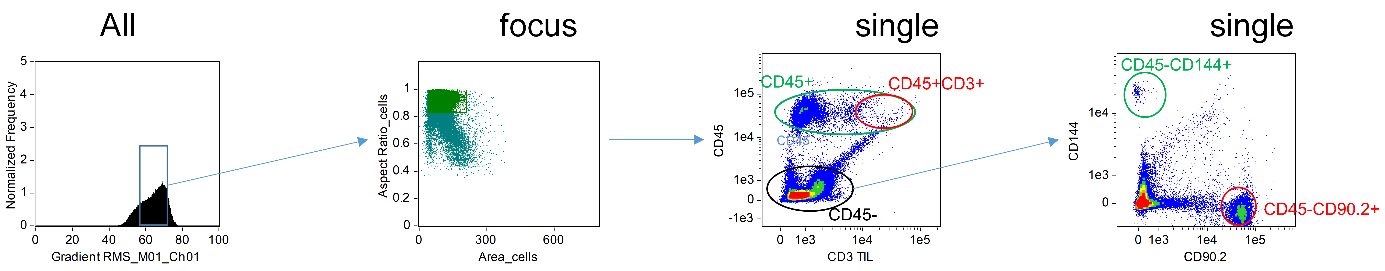


**Supplementary Figure S4. Gating strategy for TIL, CAF and TEC populations by image-based flow cytometry of single cell suspensions. a)** A representative image of collected cells, showing the entire population of cells, where cells in focus are selected upon selecting the gradient RMS and visual inspection of selected cells. **b)** Focused cells are then plotted in function of the aspect ratio and total area of the cells, where viable single cells (no debris or doublets) are selected as having an aspect ratio of > 0.8 and an area of 50-250 px. **c)** Gated viable and single cells are then plotted for the level of antibody stained CD45 (immune cells) and CD45^+^CD3^+^ (TIL marker) and the gates were selected to indicate the pure populations. **d)** From the CD45^-^ population, cells were plotted for presence of antibody stained CD144 (TEC marker) and CD90.2 (CAF marker) and the gates were selected to indicate the pure populations.


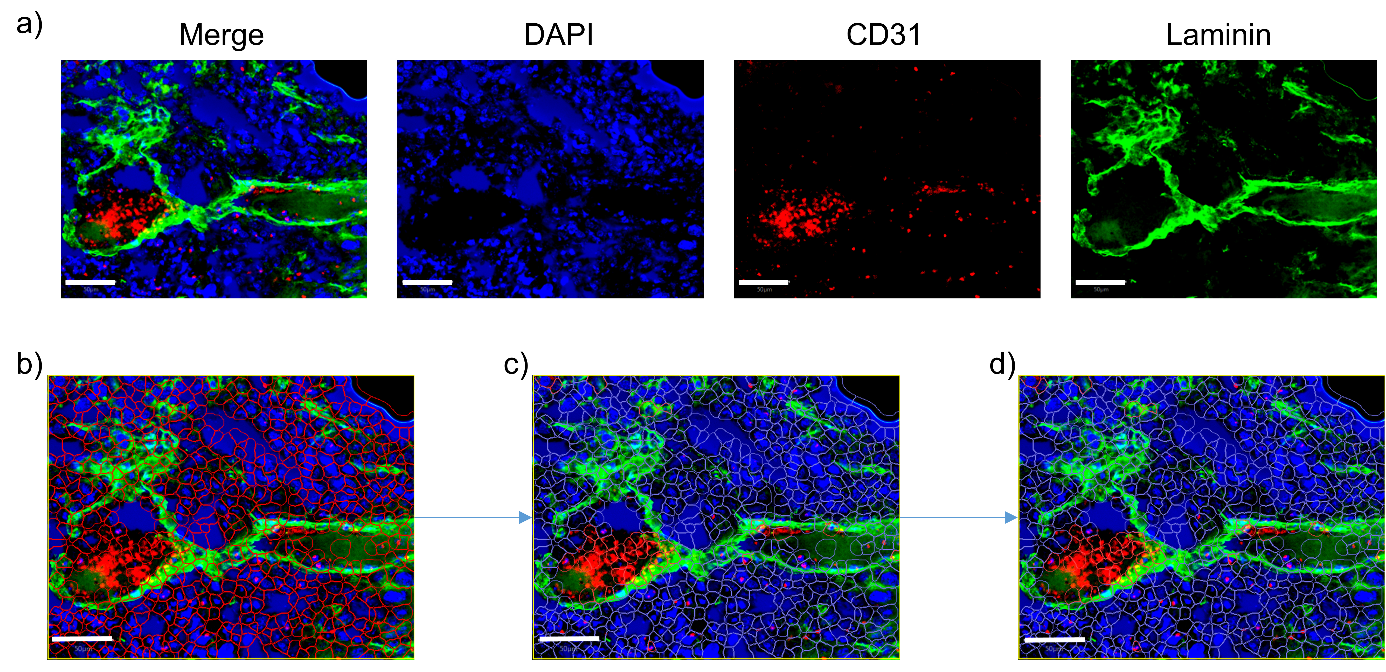


**Supplementary Figure S5. Overview of analysis strategy for detection of laminin-covered tumor-associated blood vessels in antibody-stained tissue sections using QuPath software.** **a)** A representative image of a tissue section stained for CD31 and laminin and counterstained with DAPI nuclear stain. **b)** Using DAPI nuclear stain, a threshold was selected that generated a positive selection (red selection markers) of individual cells. As DAPI only provides a nuclear stain, the area was expanded by 5 µm around all edges. **c)** Using the cell selection, a first classifier was generated, where cells positive for CD31 in the extended cytoplasm were selected (positive cells: red marking; negative cells: green marking). **d)** Using the first classifier, a second classifier can be generated, where CD31^+^ cells were evaluated for the presence of laminin. The total number of CD31^+^ cells, laminin^+^ cells, or CD31^+^ cells located next to laminin^+^ cells were then determined. The level of laminin-covered CD31^+^ cells in function of total CD31^+^ cell level is then calculated and expressed as % laminin. Scale bars: 50 µm.


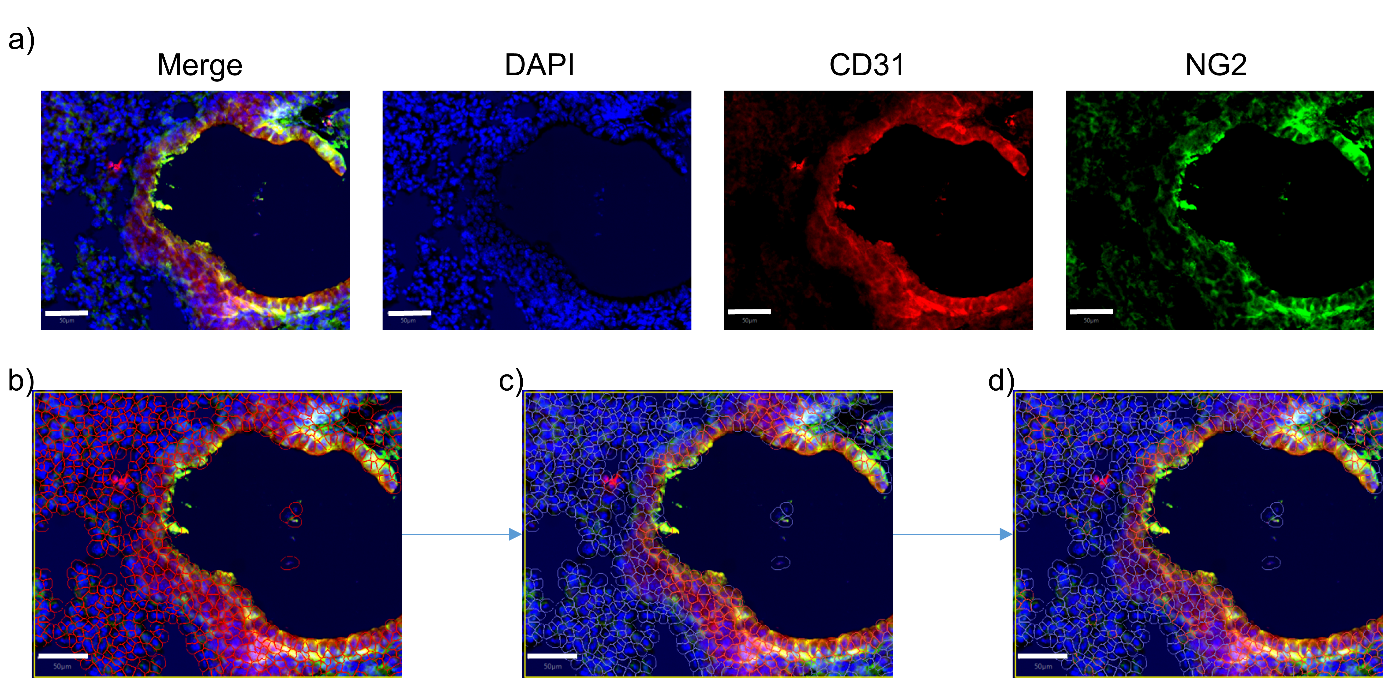


**Supplementary Figure S6. Overview of analysis strategy for detection of NG2-covered tumor-associated blood vessels in antibody-stained tissue sections using QuPath software.** **a)** A representative image of a tissue section stained for CD31 and NG2 and counterstained with DAPI nuclear stain. **b)** Using DAPI nuclear stain, a threshold was selected that generated a positive selection (red selection markers) of individual cells. As DAPI only provides a nuclear stain, the area was expanded by 5 µm around all edges. **c)** Using the cell selection, a first classifier was generated, where cells positive for CD31 in the extended cytoplasm were selected (positive cells: red marking; negative cells: green marking). **d)** Using the first classifier, a second classifier can be generated, where CD31^+^ cells were evaluated for the presence of NG2. The total number of CD31^+^ cells, NG2^+^ cells, or CD31^+^ NG2^+^ cells were then determined. The level of NG2-covered CD31^+^ cells in function of total CD31^+^ cell level is then calculated and expressed as % NG2.


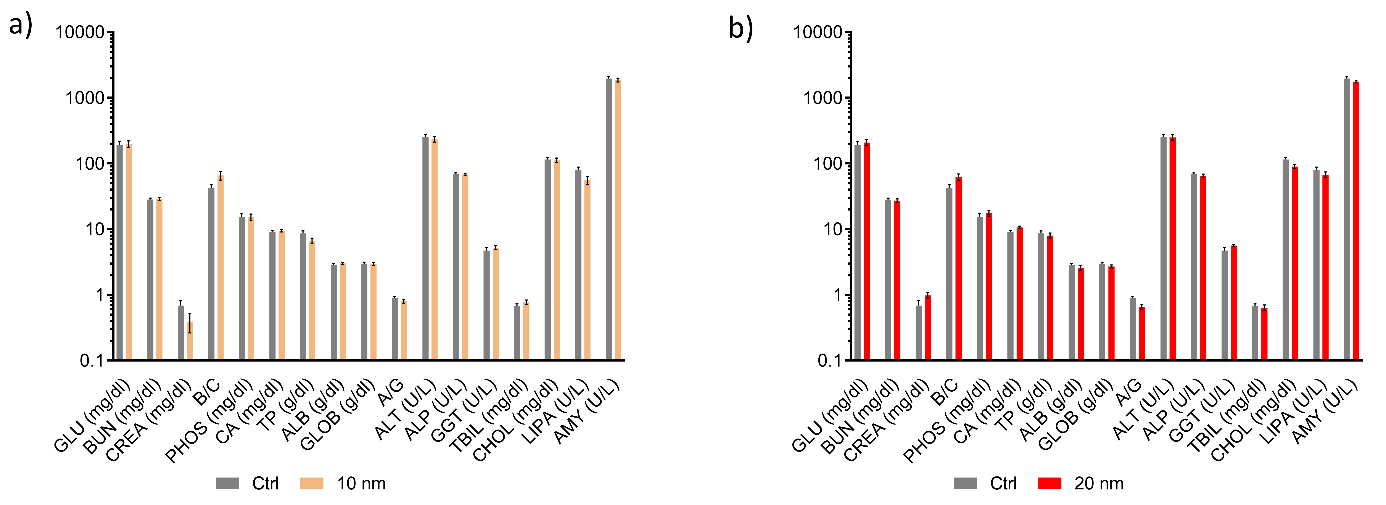


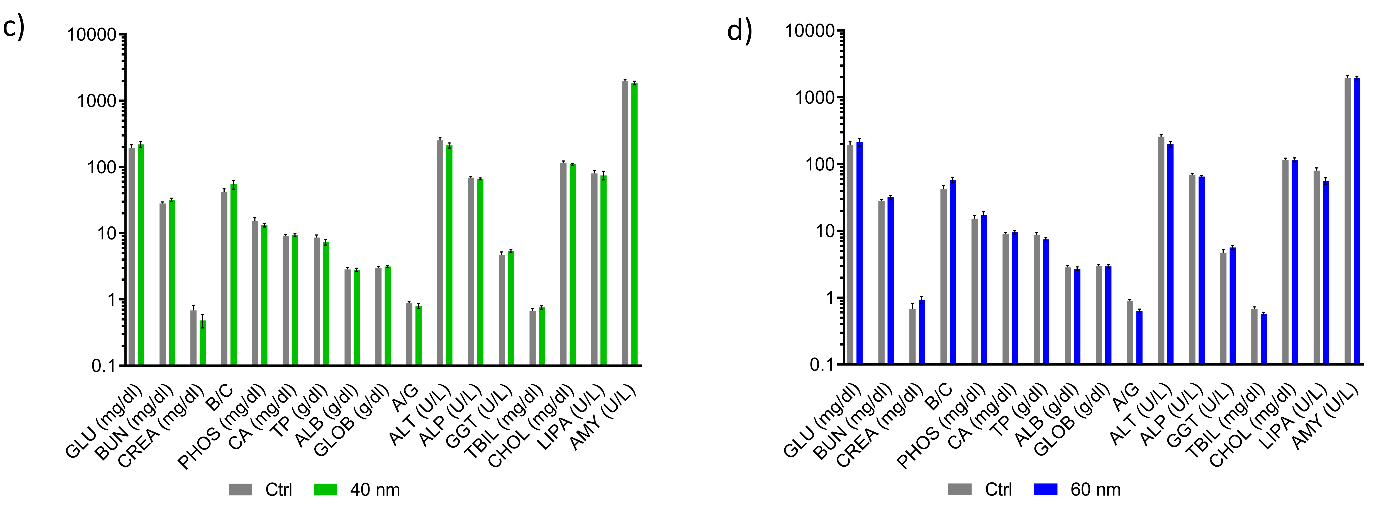


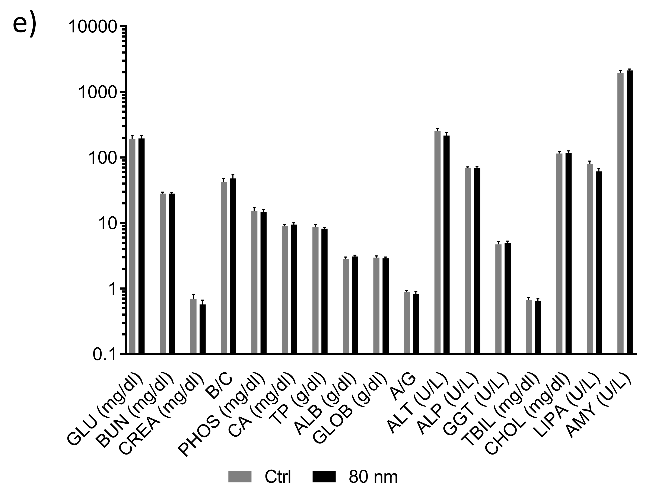


**Supplementary Figure S7. Blood biochemistry results indicate no toxicity of Au NPs.** Histograms showing blood biochemistry results of Renca-bearing control mice (light grey bars) or Renca-bearing mice exposed to **a)** Au_10_ NPs, **b)** Au_20_ NPs, **c)** Au_40_ NP, **d)** Au_60_ NPs, **e)** Au_80_ NPs. All data are expressed as mean + SEM (*n* = 6). The following markers are studied: glucose (GLU), blood urea nitrogen (BUN), creatinine (CREA), ratio of urea over creatinine (B/C), phosphates (PHOS), calcium (CA), total protein (TP), albumin (ALB), globuline (GLOB), ratio of albumin over globuline (A/G), alanine aminotransferase (ALT), alkaline phosphatase (ALP), gamma-glutamyl transpeptidase (GGT), total bilirubin (TBIL), cholesterol (CHOL), triglycerides (LIPA), alpha-amylase (AMY).


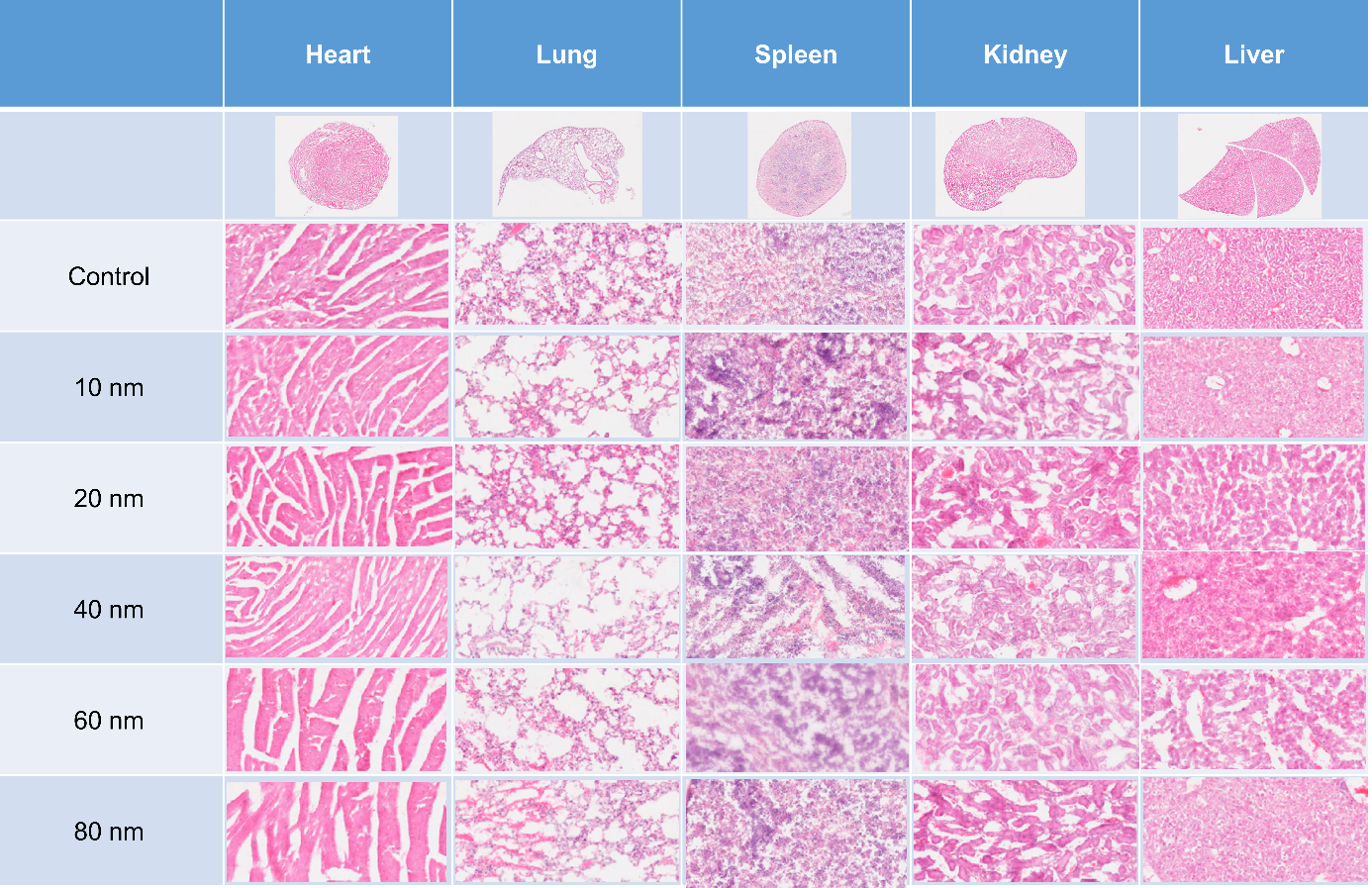


**Supplementary Figure S8. Macroscopic organ examinations do not reveal toxicity by Au NPs.** Representative H&E stained micrographs of tissue slices obtained from the heart (left column), lung (second column), spleen (middle column), kidney (4^th^ column), liver (right column). Images are shown for Renca-bearing animals that were administered PBS (top row), Au_10_ NPs (2^nd^ row), Au_20_ NPs (3^rd^ rown), Au_40_ NPs (4^th^ row), Au_60_ NPs (5^th^ row), Au_80_ NPs (bottom row).


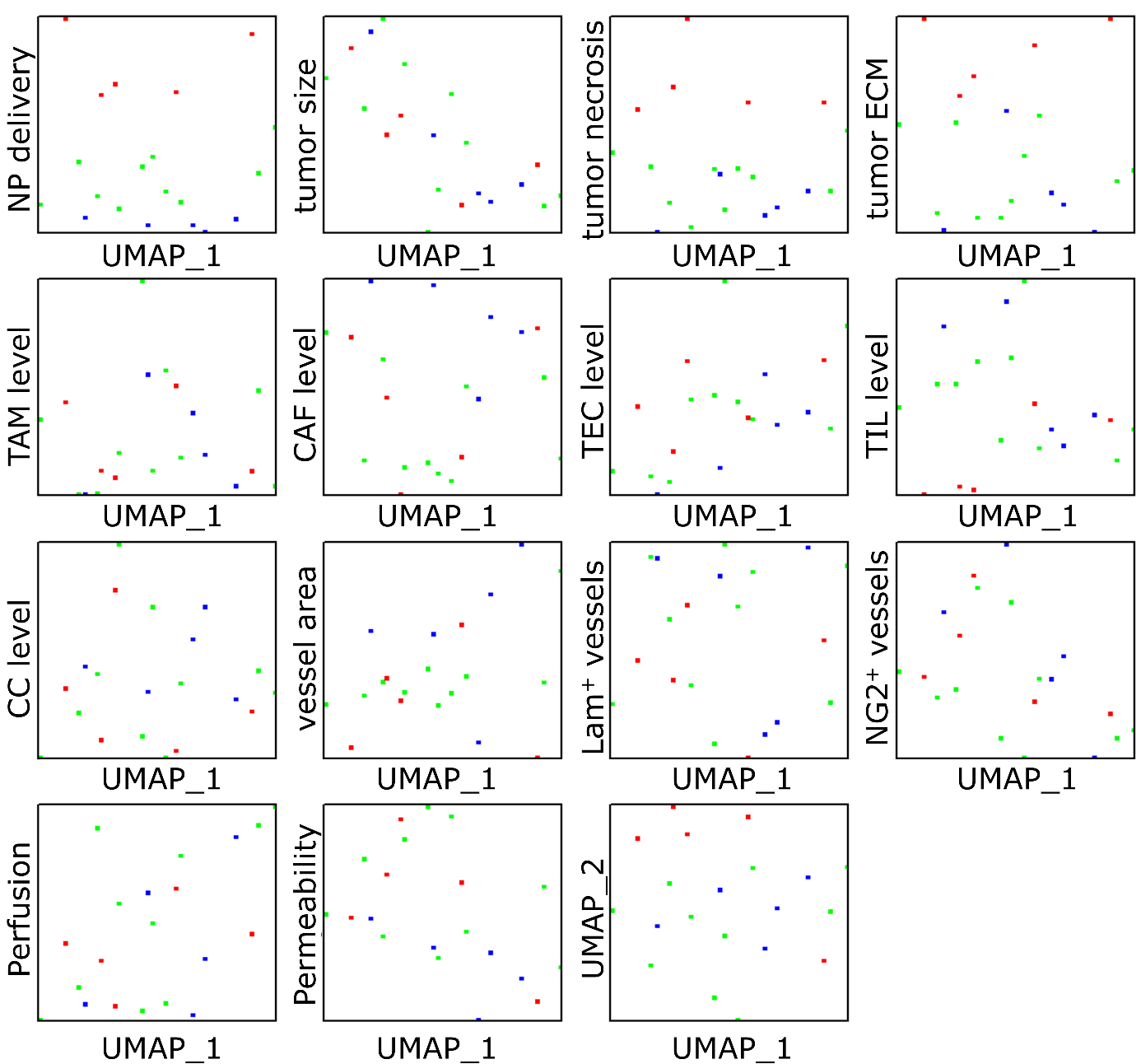


**Supplementary Figure S9.** UMAP plots for each and every tumor-associated parameter determined (as displayed in Figure 4) as a function of UMAP coordinates. For analysis, all data points for each and every animal were combined for animals receiving Au_10_ NPs. For every parameter, the values were first rescaled to a linear 0-1 scale with 0 being the lowest value for that parameter across all animals and 1 being the highest value for that parameter across all animals. The dots were colour-coded based on the upper left plot (the total tumor NP uptake level), where the 25% of animals with highest NP tumor levels were coloured red, the 25% of animals with lowest NP tumor levels were coloured blue and the remaining animals with medium NP levels were coloured green. To determine whether a particular parameter promotes or inhibits NP delivery efficacy, the red and blue groups should be separated on the Y-axis for the parameter. The parameters with most distinction were tumor necrosis and ECM.


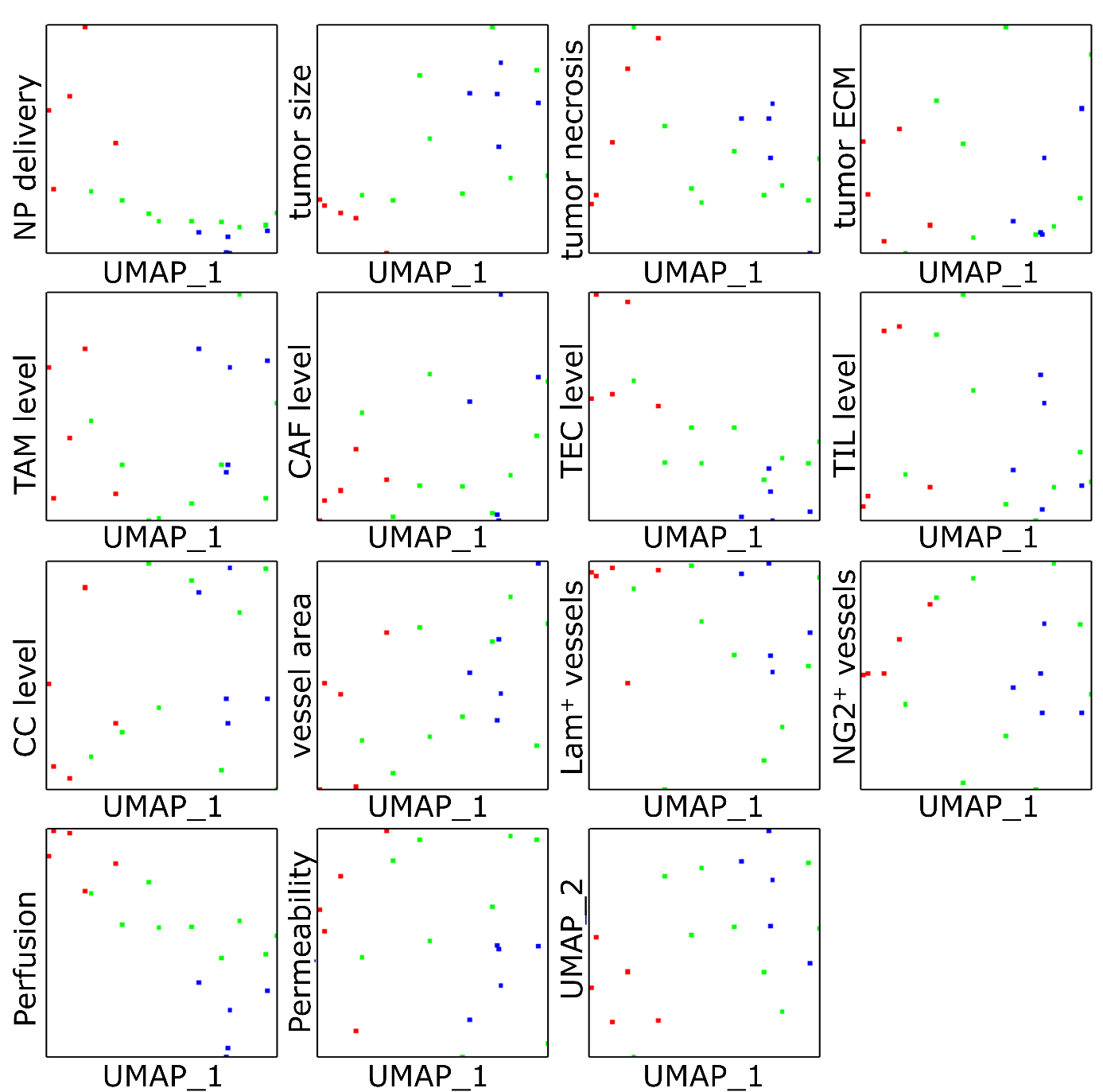


**Supplementary Figure S10.** UMAP plots for each and every tumor-associated parameter determined (as displayed in Figure 4) as a function of UMAP coordinates. For analysis, all data points for each and every animal were combined for animals receiving Au_20_ NPs. For every parameter, the values were first rescaled to a linear 0-1 scale with 0 being the lowest value for that parameter across all animals and 1 being the highest value for that parameter across all animals. The dots were colour-coded based on the upper left plot (the total tumor NP uptake level), where the 25% of animals with highest NP tumor levels were coloured red, the 25% of animals with lowest NP tumor levels were coloured blue and the remaining animals with medium NP levels were coloured green. To determine whether a particular parameter promotes or inhibits NP delivery efficacy, the red and blue groups should be separated on the Y-axis for the parameter. The parameters with most distinction were tumor size, TEC level and perfusion.


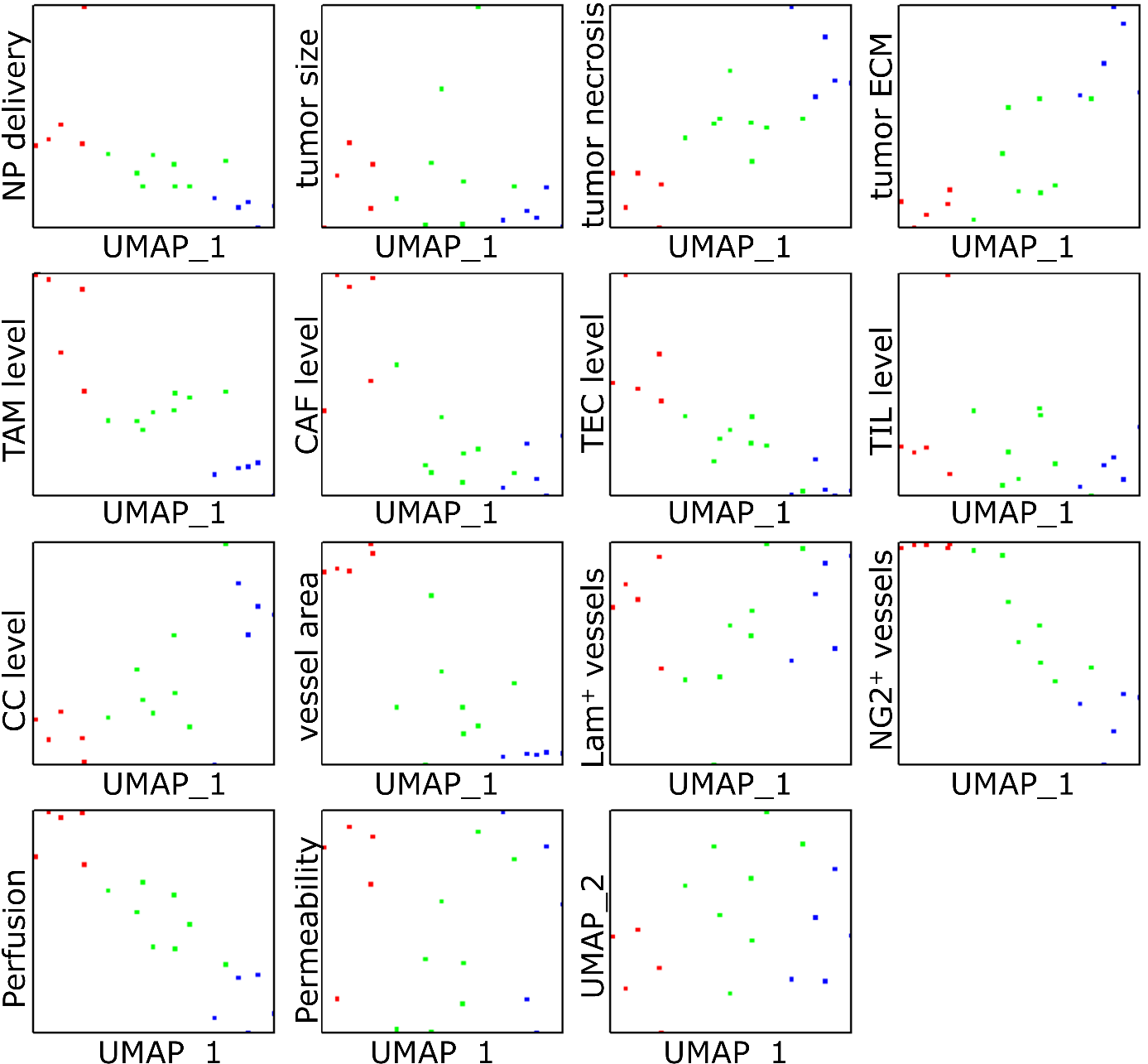


**Supplementary Figure S11.** UMAP plots for each and every tumor-associated parameter determined (as displayed in Figure 4) as a function of UMAP coordinates. For analysis, all data points for each and every animal were combined for animals receiving Au_40_ NPs. For every parameter, the values were first rescaled to a linear 0-1 scale with 0 being the lowest value for that parameter across all animals and 1 being the highest value for that parameter across all animals. The dots were colour-coded based on the upper left plot (the total tumor NP uptake level), where the 25% of animals with highest NP tumor levels were coloured red, the 25% of animals with lowest NP tumor levels were coloured blue and the remaining animals with medium NP levels were coloured green. To determine whether a particular parameter promotes or inhibits NP delivery efficacy, the red and blue groups should be separated on the Y-axis for the parameter. The parameters with most distinction were tumor necrosis, tumor ECM, TAM, CAF, TEC, CC levels, vessel area, NG2^+^ vessel levels and perfusion.


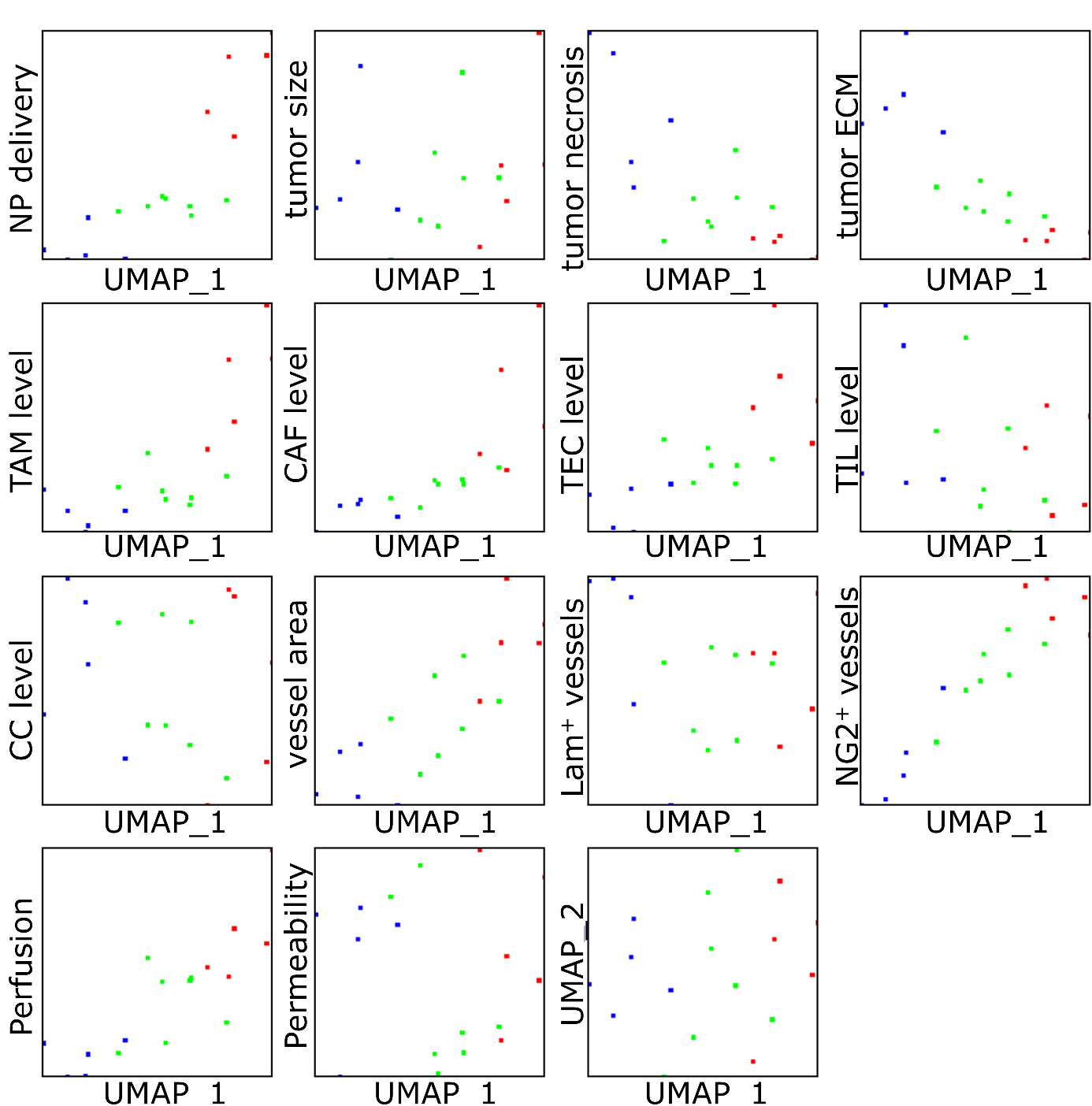


**Supplementary Figure S12.** UMAP plots for each and every tumor-associated parameter determined (as displayed in Figure 4) as a function of UMAP coordinates. For analysis, all data points for each and every animal were combined for animals receiving Au_60_ NPs. For every parameter, the values were first rescaled to a linear 0-1 scale with 0 being the lowest value for that parameter across all animals and 1 being the highest value for that parameter across all animals. The dots were colour-coded based on the upper left plot (the total tumor NP uptake level), where the 25% of animals with highest NP tumor levels were coloured red, the 25% of animals with lowest NP tumor levels were coloured blue and the remaining animals with medium NP levels were coloured green. To determine whether a particular parameter promotes or inhibits NP delivery efficacy, the red and blue groups should be separated on the Y-axis for the parameter. The parameters with most distinction were tumor necrosis, tumor ECM, TAM, CAF, TEC levels, vessel area, NG2^+^ vessel levels and perfusion.


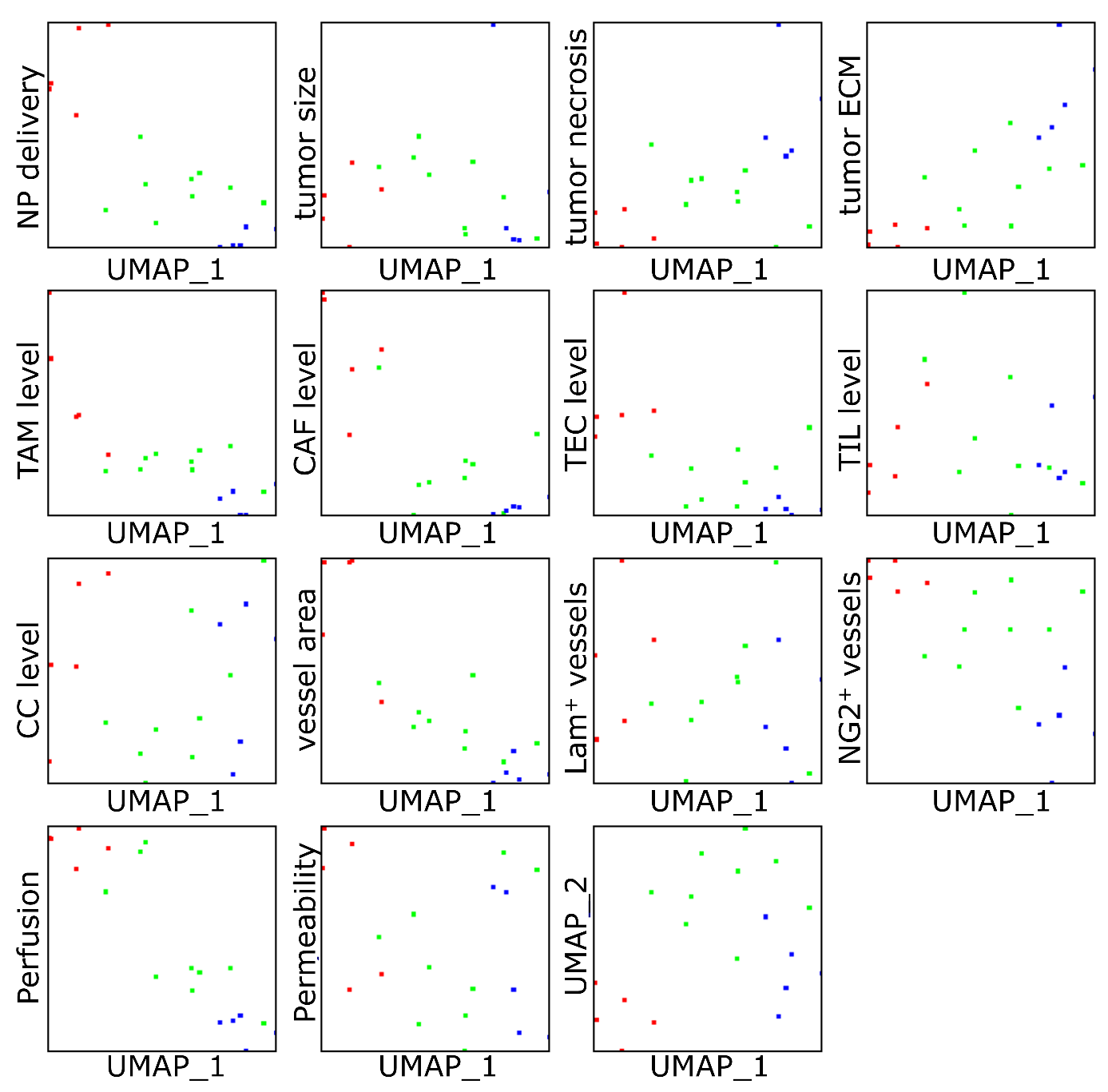


**Supplementary Figure S13.** UMAP plots for each and every tumor-associated parameter determined (as displayed in Figure 4) as a function of UMAP coordinates. For analysis, all data points for each and every animal were combined for animals receiving Au_80_ NPs. For every parameter, the values were first rescaled to a linear 0-1 scale with 0 being the lowest value for that parameter across all animals and 1 being the highest value for that parameter across all animals. The dots were colour-coded based on the upper left plot (the total tumor NP uptake level), where the 25% of animals with highest NP tumor levels were coloured red, the 25% of animals with lowest NP tumor levels were coloured blue and the remaining animals with medium NP levels were coloured green. To determine whether a particular parameter promotes or inhibits NP delivery efficacy, the red and blue groups should be separated on the Y-axis for the parameter. The parameters with most distinction were tumor necrosis, tumor ECM, TAM, CAF, TEC levels, vessel area, NG2^+^ vessel levels and perfusion.


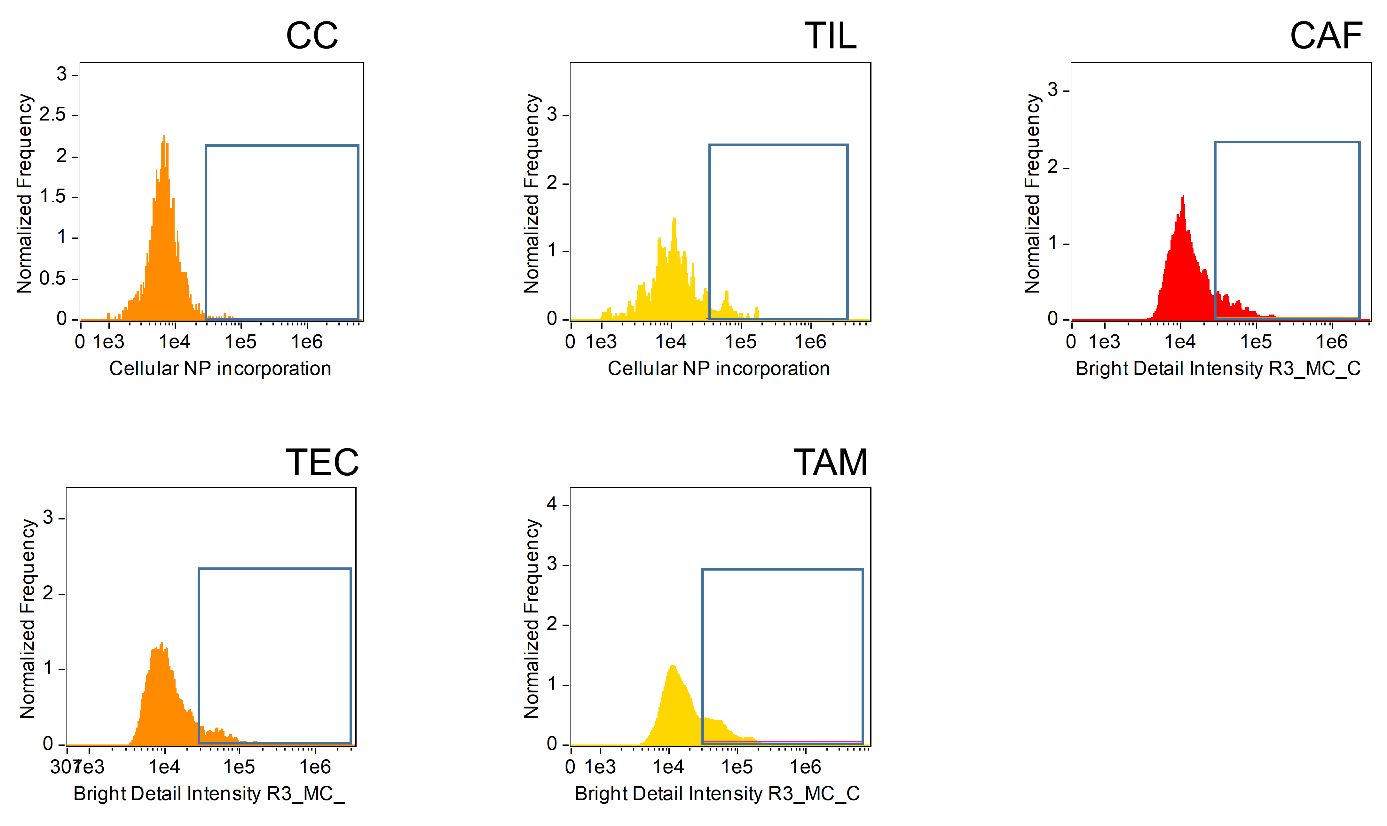


**Supplementary Figure S14. Gating strategy for NP-containing cells.** The selection of CC and TAM is illustrated in Supporting Figure S4, for TIL, CAF and TEC, this is illustrated in Supporting Figure S5. Upon selection of the gated cells, a new histogram is created that plots the Bright Detail Intensity of the masked cellular region in Ch06 (dark field channel). Only bright dots, indicating the presence of NPs, will generate positive contrast in the images. For the smaller NPs, this was validated using the corresponding fluorescence signal of the AF647-coupled NPs and overlapping spots in Ch05 and Ch06 of *in vitro* labeled cells. The threshold for detection was determined using control animals bearing Renca tumors that had never been given any NPs. The number of cells positive for NPs in the corresponding cell types can then be determined and expressed as the % of NP+ cells versus the total number of that particular cell type.


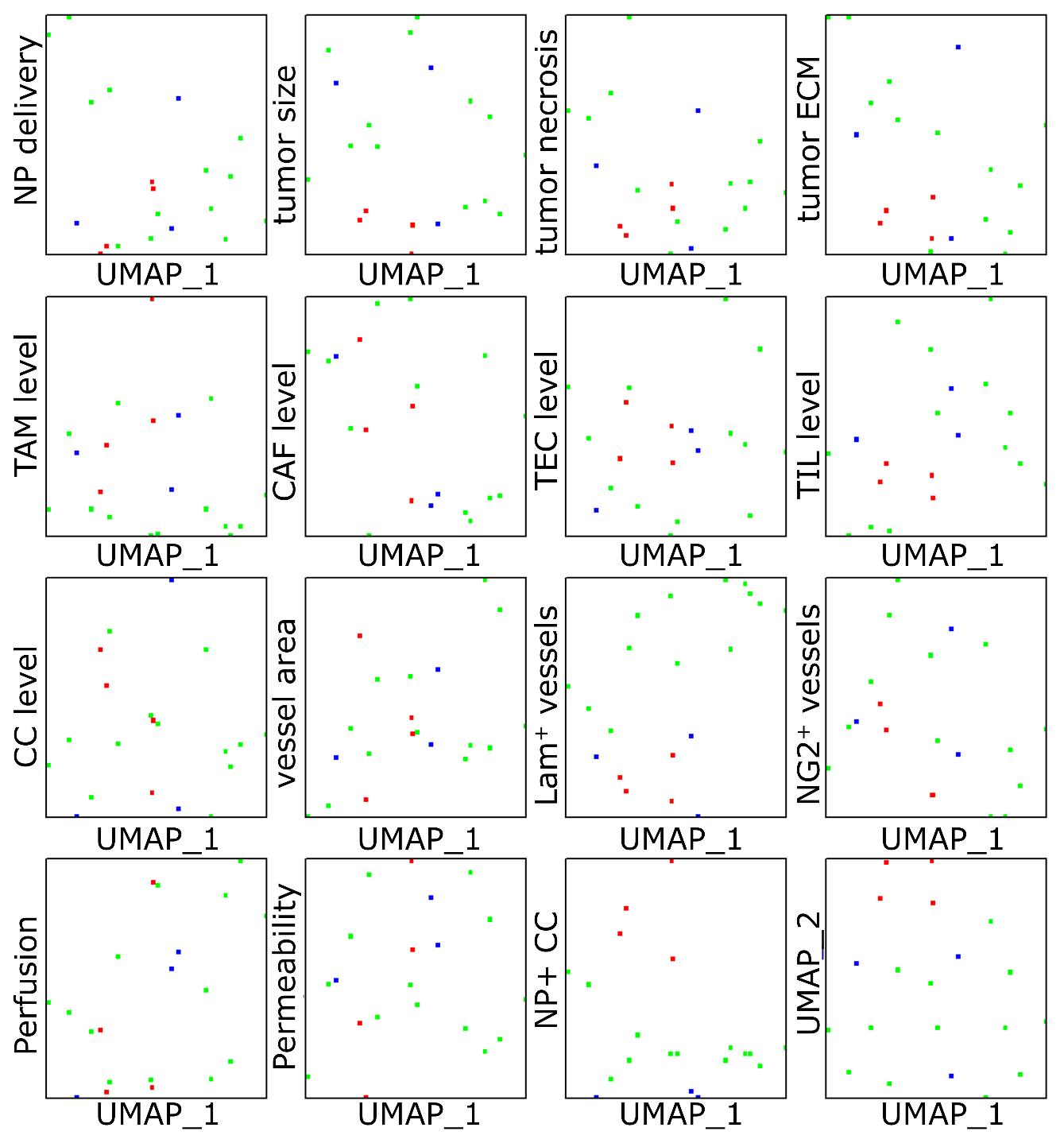


**Supplementary Figure S15.** UMAP plots for each and every tumor-associated parameter determined (as displayed in Figure 4) as a function of UMAP coordinates. For analysis, all data points for each and every animal were combined for animals receiving Au_10_ NPs. For every parameter, the values were first rescaled to a linear 0-1 scale with 0 being the lowest value for that parameter across all animals and 1 being the highest value for that parameter across all animals. The dots were colour-coded based on the level of NP^+^ cancer cells, where the 25% of animals with highest NP^+^ cancer cells were coloured red, the 25% of animals with lowest NP^+^ cancer cells were coloured blue and the remaining animals with medium NP levels were coloured green. To determine whether a particular parameter promotes or inhibits NP delivery efficacy, the red and blue groups should be separated on the Y-axis for the parameter.


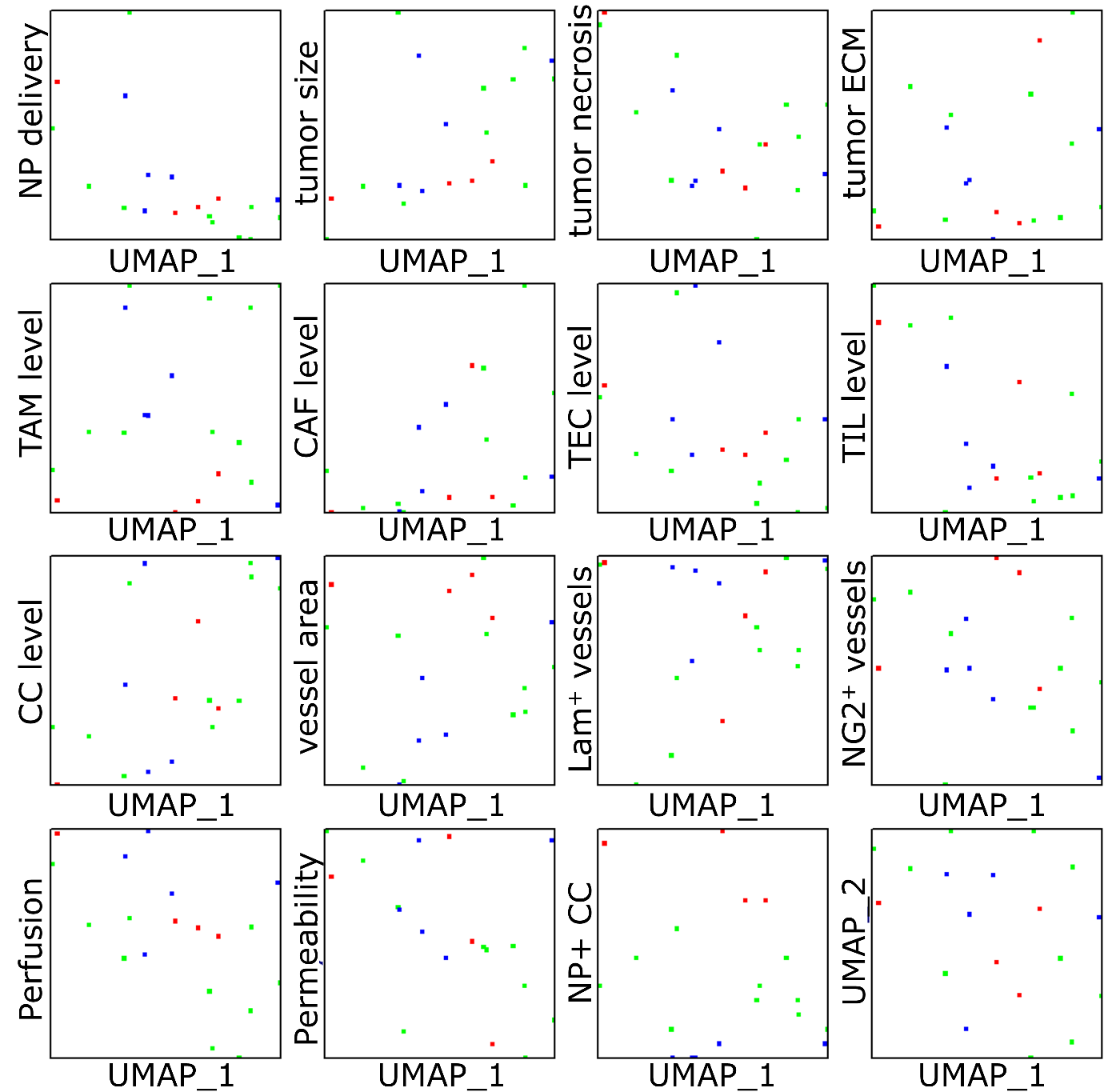


**Supplementary Figure S16.** UMAP plots for each and every tumor-associated parameter determined (as displayed in Figure 4) as a function of UMAP coordinates. For analysis, all data points for each and every animal were combined for animals receiving Au_20_ NPs. For every parameter, the values were first rescaled to a linear 0-1 scale with 0 being the lowest value for that parameter across all animals and 1 being the highest value for that parameter across all animals. The dots were colour-coded based on the level of NP^+^ cancer cells, where the 25% of animals with highest NP^+^ cancer cells were coloured red, the 25% of animals with lowest NP^+^ cancer cells were coloured blue and the remaining animals with medium NP levels were coloured green. To determine whether a particular parameter promotes or inhibits NP delivery efficacy, the red and blue groups should be separated on the Y-axis for the parameter.


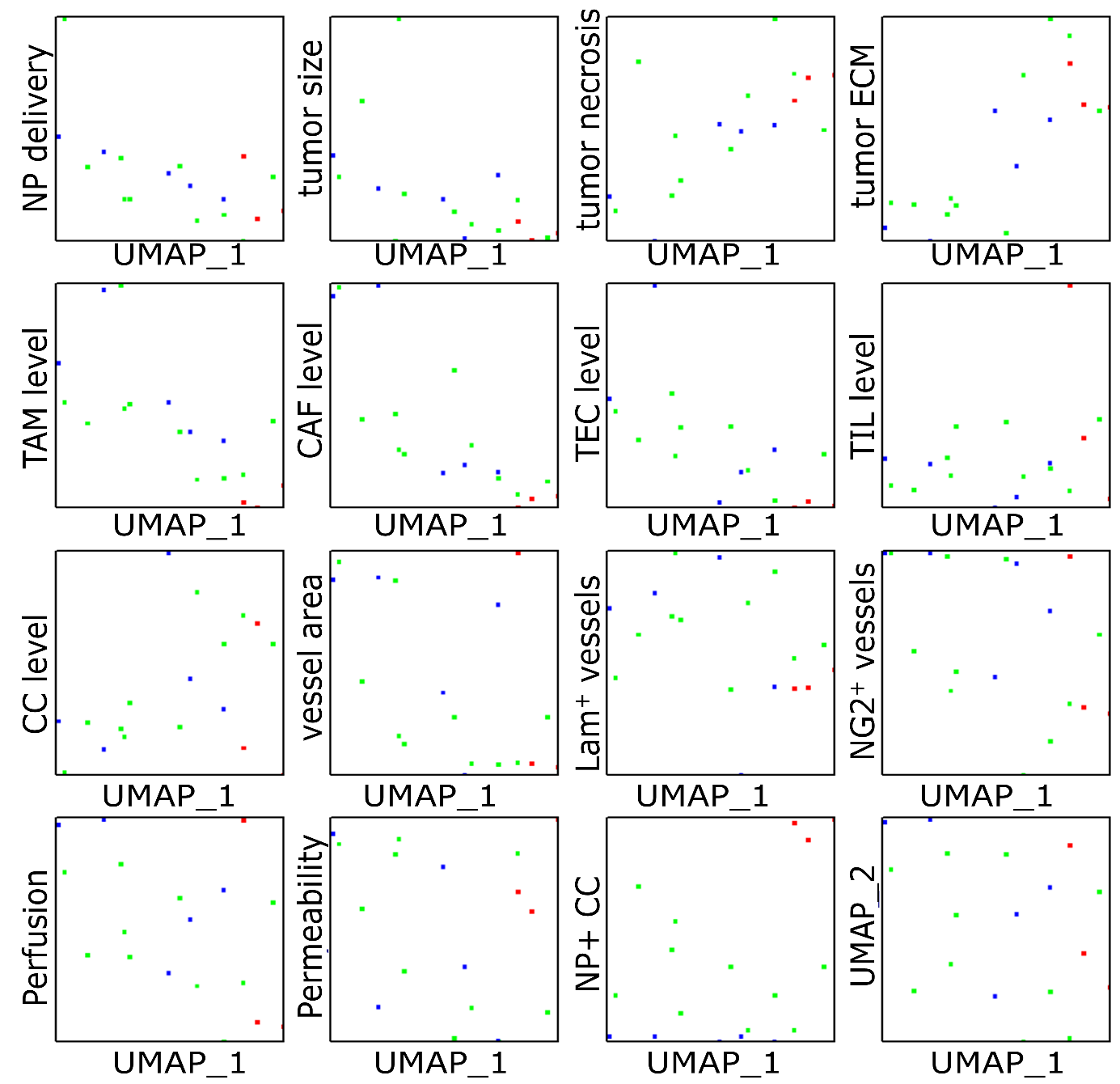


**Supplementary Figure S17.** UMAP plots for each and every tumor-associated parameter determined (as displayed in Figure 4) as a function of UMAP coordinates. For analysis, all data points for each and every animal were combined for animals receiving Au_40_ NPs. For every parameter, the values were first rescaled to a linear 0-1 scale with 0 being the lowest value for that parameter across all animals and 1 being the highest value for that parameter across all animals. The dots were colour-coded based on the level of NP^+^ cancer cells, where the 25% of animals with highest NP^+^ cancer cells were coloured red, the 25% of animals with lowest NP^+^ cancer cells were coloured blue and the remaining animals with medium NP levels were coloured green. To determine whether a particular parameter promotes or inhibits NP delivery efficacy, the red and blue groups should be separated on the Y-axis for the parameter. The most distinct parameters were TAM, CAF and TEC levels.


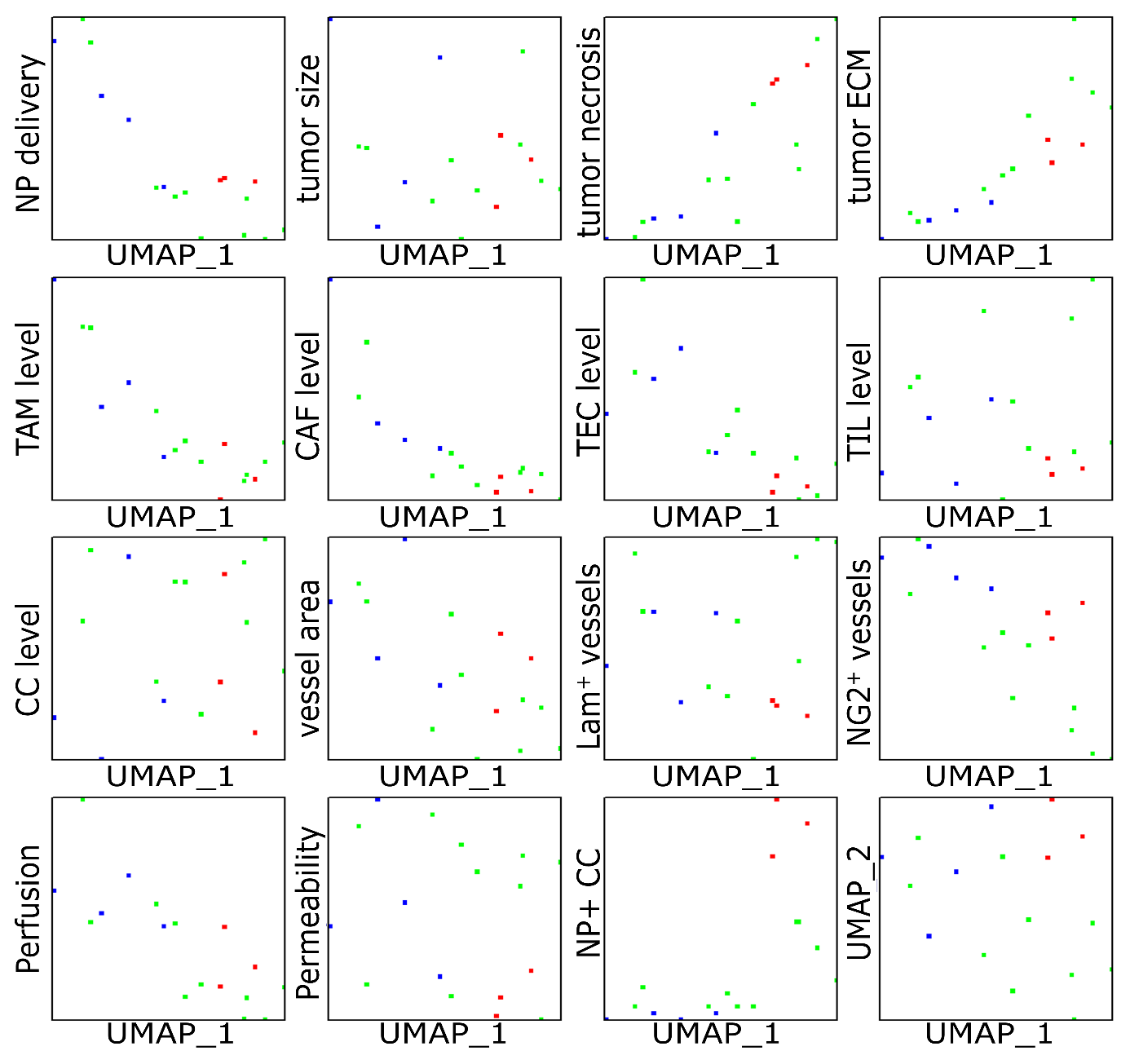


**Supplementary Figure S18.** UMAP plots for each and every tumor-associated parameter determined (as displayed in Figure 4) as a function of UMAP coordinates. For analysis, all data points for each and every animal were combined for animals receiving Au_60_ NPs. For every parameter, the values were first rescaled to a linear 0-1 scale with 0 being the lowest value for that parameter across all animals and 1 being the highest value for that parameter across all animals. The dots were colour-coded based on the level of NP^+^ cancer cells, where the 25% of animals with highest NP^+^ cancer cells were coloured red, the 25% of animals with lowest NP^+^ cancer cells were coloured blue and the remaining animals with medium NP levels were coloured green. To determine whether a particular parameter promotes or inhibits NP delivery efficacy, the red and blue groups should be separated on the Y-axis for the parameter. The most distinct parameters were TAM, CAF and TEC levels.


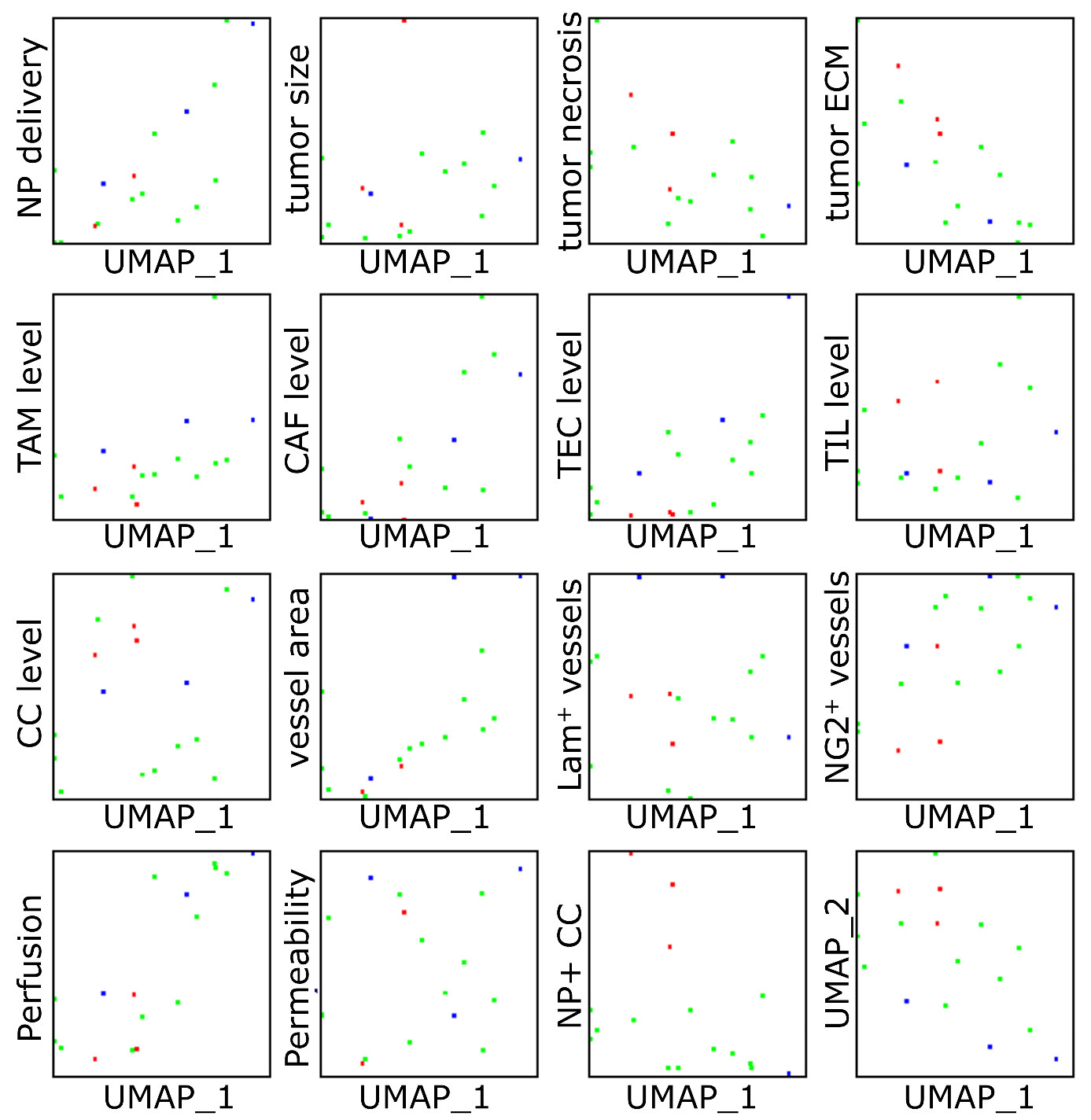


**Supplementary Figure S19.** UMAP plots for each and every tumor-associated parameter determined (as displayed in Figure 4) as a function of UMAP coordinates. For analysis, all data points for each and every animal were combined for animals receiving Au_40_ NPs. For every parameter, the values were first rescaled to a linear 0-1 scale with 0 being the lowest value for that parameter across all animals and 1 being the highest value for that parameter across all animals. The dots were colour-coded based on the level of NP^+^ cancer cells, where the 25% of animals with highest NP^+^ cancer cells were coloured red, the 25% of animals with lowest NP^+^ cancer cells were coloured blue and the remaining animals with medium NP levels were coloured green. To determine whether a particular parameter promotes or inhibits NP delivery efficacy, the red and blue groups should be separated on the Y-axis for the parameter. The most distinct parameters were TAM, CAF and TEC levels.
